# Development and external validation of an interpretable machine learning model for predicting prolonged postoperative ICU length of stay in coronary artery bypass grafting patients using MIMIC-IV 3.1 and eICU-CRD 2.0

**DOI:** 10.1186/s12911-026-03659-y

**Published:** 2026-06-27

**Authors:** Dayan Liu, Pengyu Lu, Yulan Meng, Xianglong Liu, Wei Huang

**Affiliations:** 1 Department of Anesthesiology, Kaifeng Central Hospital, Kaifeng, Henan, 475000 China; 2 Department of Critical Care Medicine, Tacheng People’s Hospital of Yili Kazak Autonomous Prefecture, Xinjiang Uygur Autonomous Region, Tacheng, 834300 China; 3 Department of Medical Imaging, The Third Affiliated Hospital of Zhengzhou University Zhengzhou, Henan, 450000 China; 4 Department of Critical Care Medicine, First Hospital of Dalian Medical University, Dalian, Liaoning 116012 China

**Keywords:** Coronary artery bypass grafting, Intensive care unit length of stay, Fluid management, Charlson comorbidity index, Atrial fibrillation, Machine learning, Predictive modeling, SHAP analysis

## Abstract

**Background:**

Prolonged postoperative intensive care unit (ICU) length of stay (LOS) after coronary artery bypass grafting (CABG) drives resource use yet remains difficult to anticipate at the 24-hour ICU evaluation moment when extended monitoring beyond 72 h is first considered. We developed and externally validated an interpretable machine-learning decision-support calculator for this deployment moment.

**Methods:**

Adult CABG patients were identified from MIMIC-IV 3.1 (*n* = 6,919; 7:3 stratified split for development) and eICU-CRD 2.0 (*n* = 5,972; external validation). The outcome was prolonged ICU LOS (> 3 days). Elastic Net plus Boruta selected eight bedside features collected within the first 24 h of ICU admission: 24-hour fluid intake, Charlson Comorbidity Index (CCI), Sequential Organ Failure Assessment (SOFA) score, Simplified Acute Physiology Score II (SAPS-II), Glasgow Coma Scale (GCS), vasopressor use, congestive heart failure, and atrial fibrillation. Nine machine-learning algorithms were compared by 10-fold cross-validation paired t-tests with Bonferroni and Benjamini–Hochberg correction. Calibration metrics included Hosmer–Lemeshow test, Integrated Calibration Index (ICI), expected-to-observed ratio, intercept, and slope (full Methods). SHapley Additive exPlanations (SHAP) provided per-patient feature attribution.

**Results:**

CatBoost was selected as the deployed model. On the MIMIC-IV internal test set (*n* = 2,076), the area under the receiver-operating-characteristic curve (AUC) was 0.7739 (95% confidence interval 0.7379–0.8099), Hosmer–Lemeshow *p* = 0.224, calibration slope 0.973. On the eICU-CRD external cohort, AUC was 0.6452 (95% CI 0.6311–0.6602), calibration slope 0.998, ICI 0.023. At the prevalence-anchored threshold of 0.30, sensitivity was 0.55, specificity 0.65, positive predictive value 0.40, and negative predictive value 0.77. Decision Curve Analysis showed positive net benefit over treat-all and treat-none across t = 0.20–0.40. SHAP top-3 features were 24-hour fluid intake, CCI, and atrial fibrillation.

**Conclusions:**

The model provides decision support at the post-CABG 24-hour ICU evaluation moment with modest discrimination and well-calibrated probabilities at deployment. The deployed online calculator renders per-patient feature contributions transparent at the bedside via SHAP; prospective validation is required before clinical deployment.

**Supplementary Information:**

The online version contains supplementary material available at https://doi.org/10.1186/s12911-026-03659-y.

## Introduction

Coronary artery bypass grafting (CABG) is a widely adopted surgical intervention for coronary artery disease; however, patients remain at risk for serious postoperative complications including cardiac dysfunction, renal insufficiency, sepsis, pulmonary infection, and mortality. The occurrence of these complications invariably results in prolonged intensive care unit (ICU) length of stay (LOS) and substantially increased healthcare costs [1, 2]. Thus, postoperative ICU LOS represents a critical outcome measure reflecting surgical success and is a significant predictor of overall patient prognosis and resource utilization. Accurate prediction of ICU LOS would enable improved patient risk stratification and facilitate more efficient healthcare resource planning.

Machine learning (ML) has emerged as a transformative approach in developing sophisticated clinical predictive models, with increasingly demonstrated applications across surgical specialties and critical care medicine [3, 4]. In this study the specific objectives were to: (1) identify critical clinical risk factors associated with prolonged postoperative ICU length of stay (LOS) in CABG patients; (2) develop and validate a robust machine learning-based predictive model through comprehensive algorithmic comparison; (3) enhance model transparency and clinical interpretability through SHAP (SHapley Additive exPlanations) analysis; (4) translate model findings into a practical, implementable clinical decision support tool. The tool is positioned as a decision-support calculator at the 24-hour ICU evaluation moment, complementing preoperative risk stratification rather than competing with it; a direct head-to-head comparison with a preoperative reference model on the eICU external cohort is reported in the Discussion.

## Materials and methods

### Data sources and retrieval

MIMIC-IV 3.1 is a large database developed and maintained by the Computational Physiology Laboratory at the Massachusetts Institute of Technology, containing medical information from patients admitted to the intensive care unit (ICU) of Beth Israel Deaconess Medical Center [5]. The eICU Collaborative Research Database 2.0 (eICU-CRD 2.0) is a publicly available, multi-center ICU database developed through collaboration between the MIT Computational Physiology Laboratory and Philips eICU Research Institute, comprising data from over 200,000 patients across 208 hospitals in the United States [6]. The authors (Pengyu Lu) obtained access permissions to these databases (CITI certificate number: 13605560). Both MIMIC-IV 3.1 and eICU-CRD 2.0 databases are de-identified and do not contain patient privacy information; therefore, the requirement for informed consent was waived.

PostgreSQL 16.4.1 and Navicat 16.3.5 software were used to execute Structured Query Language (SQL) queries to retrieve CABG patient data from the MIMIC-IV 3.1 database (ICD-9 code: “361%”, ICD-10 code: “021%”) for the period from January 1, 2008 to December 31, 2022. From the eICU-CRD 2.0 database, adult CABG patient data with diagnostic strings containing “Coronary artery bypass” or “CABG” were retrieved for the period from January 1, 2014 to December 31, 2015. Inclusion criteria were, as implemented in the cohort SQL: (1) age ≥ 18 years; (2) hospital LOS ≥ 24 h; (3) admission to an ICU during the index hospitalisation with ICU stay > 0; (4) only the first CABG procedure was retained for patients with multiple records. Exclusion criteria were: (1) age < 18 years; (2) hospital LOS < 24 h. We note explicitly that ICU LOS ≥ 24 h was not a separate inclusion criterion; the cohort therefore contains 324 patients (4.7%) whose ICU LOS was shorter than 24 h and whose 24-hour feature values were drawn from whatever ICU time was available, supplemented by imputation where needed. The implications of this cohort-definition choice are discussed in the Limitations subsection “Cohort definition and short-stay patients”.

For the included patients, 64 variables were extracted: demographics; vital signs, routine blood tests, liver and renal function panels, fluid balance, and vasoactive medication use within the first 24 h of ICU admission; duration of invasive mechanical ventilation; five severity scores — Charlson Comorbidity Index (CCI), Sequential Organ Failure Assessment (SOFA), Simplified Acute Physiology Score II (SAPS-II), Oxford Acute Severity of Illness Score (OASIS), and Glasgow Coma Scale (GCS); number of bypass grafts; admission comorbidities (myocardial infarction, congestive heart failure, peripheral vascular disease, cerebrovascular disease, diabetes, renal disease, chronic obstructive pulmonary disease, atrial fibrillation, hypertension, and chronic kidney disease); and post-operative ICU LOS. Patients were stratified into non-prolonged (≤ 3 days) and prolonged (> 3 days) groups. ICU LOS distributions were right-skewed (MIMIC-IV median 1.95 days, mean 3.06 days; eICU-CRD median 2.01 days, mean 3.00 days), so the median is the appropriate descriptive statistic. The > 3-day cutpoint was retained on three grounds independent of the mean: (i) it is widely used in the cardiac-surgery and ICU literature as a clinically-anchored marker of resource use and discharge readiness; (ii) deployment at the 24-hour post-CABG ICU evaluation moment makes > 3 days the operationally relevant threshold for continued ICU-level monitoring beyond the standard 72-hour window; and (iii) at approximately 30% prevalence the > 3-day outcome is more balanced for ML training than the ≈ 12% prevalence at > 5 days. A > 5-day sensitivity analysis is reported in the Limitations section and Additional file 1 (Supplementary Material 7, Table S13). Two related sensitivity analyses — an hourly fluid_input trajectory check and a 6-/12-/24-hour feature-window re-training — are pre-specified in Section “General characteristics of the study population” and reported in Additional file 1 (Supplementary Material 7, Tables S7–S8 and Figure S17).

### Statistical methods

Reporting follows the TRIPOD + AI 2024 guideline for clinical prediction models that use regression or machine learning methods [7]. SPSS version 25.0 software was used for data analysis. The Kolmogorov-Smirnov test was applied to assess data distribution. For normally distributed data, independent two-sample t-tests were used to compare between groups, with results expressed as mean and standard deviation. For non-normally distributed data, the Mann-Whitney U test was employed, with results expressed as median and interquartile range (IQR). Categorical variables were expressed as percentages, and chi-square tests were used for group comparisons.

All subsequent data processing steps were implemented using Python 3.8 with a global random seed set to 42 to ensure reproducibility of results.

#### Data set establishment

The MIMIC-IV 3.1 dataset served as the model development dataset and was split in a 7:3 ratio into training and internal validation datasets. The eICU-CRD 2.0 dataset served as an independent external validation dataset.

#### Data preprocessing

Features with missing values exceeding 30% in the MIMIC-IV 3.1 dataset were excluded; remaining missing data were handled by Multivariate Imputation by Chained Equations (MICE) — continuous variables standardized then iteratively imputed via random forest regression (≤ 10 iterations), categorical variables imputed sequentially via random forest classifiers ordered by missing rate. Imputation models were trained exclusively on the training set to prevent data leakage and applied to the validation and external validation cohorts. Per-variable diagnostics comprised the Kolmogorov–Smirnov (KS) two-sample test, median and mean bias, and the variance ratio for continuous variables, and chi-square tests for categorical variables; a composite Imputation Quality Global Index (IQGI) was computed for descriptive purposes only, not as a per-variable adequacy criterion. Variables failing the KS test (*p* < 0.05) are reported individually in the Results, and three imputation sensitivity analyses (complete-case; predictive mean matching, m = 5; missForest) verified robustness (Additional file 1: Supplementary Material 7, Tables S5–S6). Two further pre-specified sensitivity analyses addressed feature-window concerns. First, hourly cumulative fluid_input was re-extracted from mimiciv_icu.inputevents over the first 24 ICU hours and stratified by outcome (Additional file 1: Supplementary Material 7, Table S8 and Figure S17). Second, full-pipeline CatBoost was re-trained at 6-, 12-, and 24-hour feature windows with windowed SOFA/GCS recomputed from mimiciv_derived (SAPS-II retained at first-day value; eICU re-extraction limited to fluid_input); absolute AUCs from this separate re-extraction pipeline are confirmatory rather than primary and are not directly comparable to the principal external AUC reported in Section “Internal and external validation of the optimal model” (Additional file 1: Supplementary Material 7, Table S7). The eICU-CRD 2.0 dataset served as the independent external validation cohort.

#### Feature selection

A dual-stage feature selection strategy was employed to systematically identify clinically relevant and statistically significant predictive variables from the candidate pool. In the first stage, Elastic Net regression was applied, with L1/L2 mixture ratios (range: 0.1-1.0) optimized through 10-fold cross-validation. This approach leverages coefficient shrinkage mechanisms to identify non-zero coefficient features within a logistic regression framework, with the top 10 ranked features retained. In the second stage, the Boruta algorithm was applied based on statistical comparison between random forest importance scores and shadow feature importance (α = 0.05), employing 400 iterations to confirm features with genuine predictive power, again retaining the top 10 ranked features. The Borda weighted voting method was subsequently used to integrate results from both algorithms. Core features confirmed by both methods were identified as consensus predictive factors. Additional high-scoring features from either method were considered for supplementation using Borda scores. Final feature selection balanced model robustness with clinical practicality and bedside feasibility, resulting in a parsimonious set of 8 features for model development.

#### Model construction

Hyperparameter optimization for each algorithm was conducted through exhaustive Grid Search Cross-Validation (GridSearch CV), identifying optimal hyperparameter combinations. Each candidate model was trained and rigorously evaluated using 10-fold nested cross-validation on the training dataset to provide robust estimates of generalization performance while preventing overfitting. Nine distinct ML algorithms were evaluated, representing diverse modeling paradigms: Logistic Regression (LR), Random Forest (RF), Support Vector Machine (SVM), K-Nearest Neighbors (KNN), eXtreme Gradient Boosting (XGBoost), Decision Tree (DT), Deep Neural Network (DNN), Categorical Boosting (CatBoost), and Light Gradient Boosting Machine (LightGBM). This comprehensive evaluation strategy enabled fair algorithmic comparison and selection of the optimal approach for clinical application.

#### Optimal prediction model selection and validation

Model selection was based on 10-fold cross-validation AUC, standard deviation, and paired t-test contrasts among the nine candidate algorithms on the training dataset. Multiplicity over the 36 pairwise comparisons was controlled with two complementary procedures: the Bonferroni correction [8] (per-test threshold = 0.05 / 36 ≈ 0.0014) and the Benjamini–Hochberg false discovery rate procedure [9] (BH-FDR, q = 0.05). Exact two-sided p-values were computed against 10-fold cross-validation degrees of freedom (df = 9); uncorrected, Bonferroni-adjusted, and BH-FDR–adjusted results are reported in parallel. The selected model underwent internal and external validation. Calibration assessment included Brier score, Hosmer-Lemeshow goodness-of-fit (decile-based; χ² and p), Integrated Calibration Index (ICI; mean absolute deviation between observed and predicted probability across 20 bins), expected-to-observed (E/O) ratio, and calibration intercept and slope (estimated by logistic regression of the binary outcome on the linear predictor). Discrimination was reported via ROC and PR-AUC curves; clinical utility via Decision Curve Analysis (DCA). Multicollinearity among the deployed features was assessed via Variance Inflation Factor (VIF), with reduced-model comparisons (severity-score block versus raw-variable block) trained under identical pipeline parameters. For the external cohort we additionally applied 5-fold cross-validated logistic recalibration (intercept + slope only) and report uncalibrated and recalibrated metrics in parallel; this transformation does not alter rank order and therefore leaves AUC unchanged but corrects systematic over- or under-prediction.

#### Optimal model interpretability analysis and clinical decision support tool development

To address the interpretability challenge inherent in machine learning, SHapley Additive exPlanations (SHAP) analysis was employed to provide model-agnostic explanations of individual predictions. An interactive web-based risk calculator was developed to translate model outputs into a practical clinical decision support tool for real-time risk assessment.

## Results

### General characteristics of the study population

**Fig. 1:**
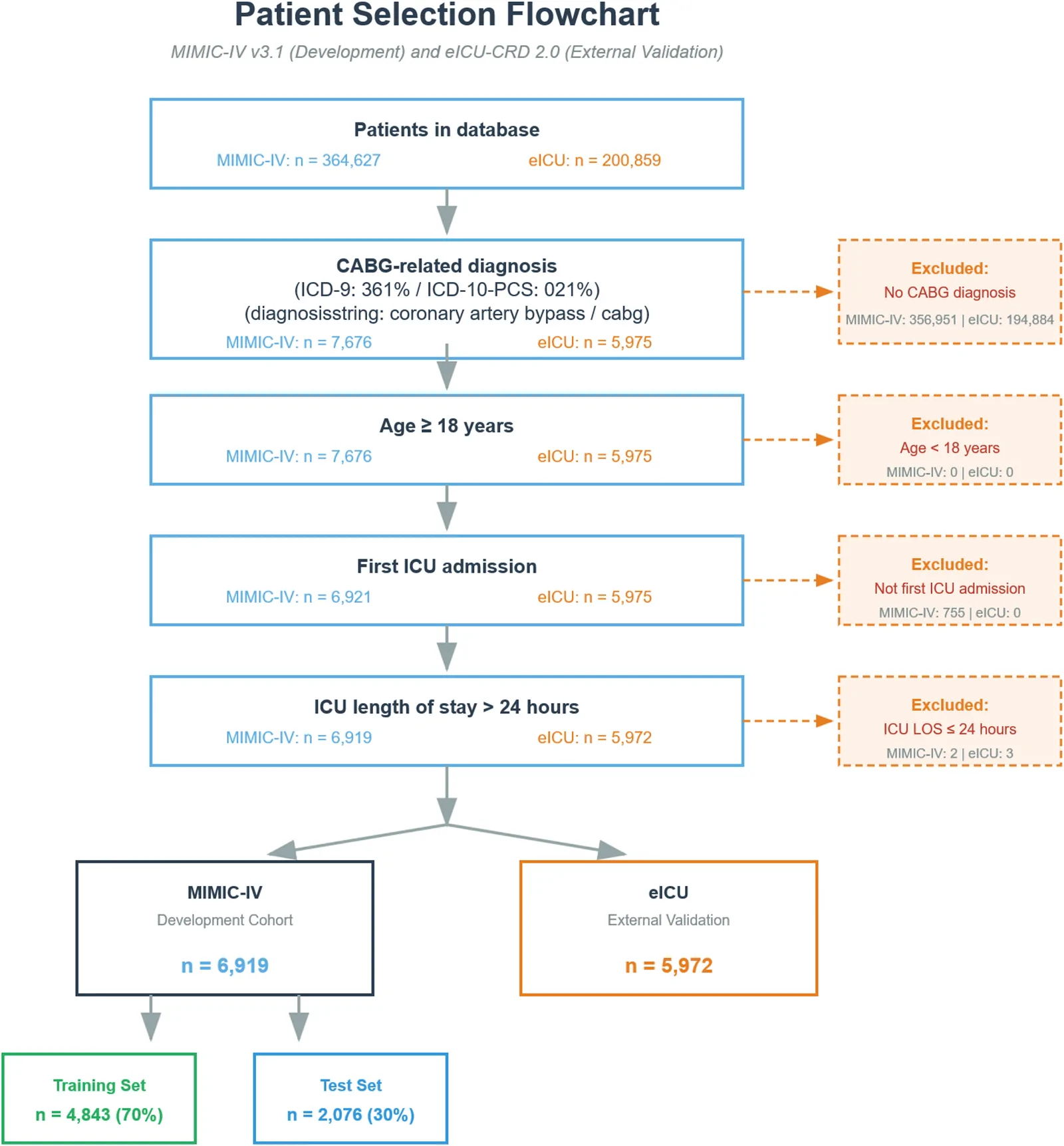
Flowchart of patient screening for the prediction model of ICU length of stay after CABG surgery

A total of 6,919 CABG patients meeting inclusion criteria were retrieved from the MIMIC-IV 3.1 database (2008–2022), with a mean age of 67.2 years (median 68 years), and 5,389 male patients (77.9%). Patients were randomly stratified in a 7:3 ratio into the training dataset (*n* = 4,843) and internal validation dataset (*n* = 2,076). In the training dataset, 3,405 patients (70.3%) had postoperative ICU LOS ≤ 3 days (non-prolonged ICU stay group), and 1,438 patients (29.7%) had LOS > 3 days (prolonged ICU stay group). In the internal validation dataset, the two groups comprised 1,459 (70.3%) and 617 (29.7%) patients, respectively. From the eICU-CRD 2.0 database, a total of 5,972 CABG patients meeting inclusion criteria were identified for the period from 2014 to 2015. The overall cohort had 4,442 male patients (74.4%). The non-prolonged ICU stay group comprised 4,196 patients (70.3%), while the prolonged ICU stay group comprised 1,776 patients (29.7%). The complete patient selection and stratification process is illustrated in Fig. 1.

The comparison of baseline characteristics and statistical differences for the cohort are shown in Supplementary Material 1, Table S1. Nine features with missing values exceeding 30% were excluded from the original 64 variables, including albumin (91.9% missing), troponin (93.1% missing), N-terminal B-type natriuretic peptide (99.6% missing), alanine aminotransferase (86.6% missing), aspartate aminotransferase (86.6% missing), alkaline phosphatase (87.1% missing), total bilirubin (86.5% missing), lactate dehydrogenase (91.2% missing), and blood product input (76.2% missing). Consequently, 55 variables were retained for subsequent feature selection in predictive model construction.

### Assessment of data imputation quality

The MICE imputation algorithm was evaluated using per-variable diagnostics. Of 29 imputed variables, 26 satisfied KS distributional consistency (*p* > 0.05). Three variables did not pass and merit explicit disclosure: fluid_input within 24 h (12.08% missing; KS *p* = 0.0002; median bias 13.76%), temperature (10.72% missing; KS *p* = 0.0011), and invasive ventilation hours (22.07% missing; KS *p* < 0.001; median bias 25.17%). The composite IQGI score of 95.2 reflects aggregate performance dominated by the 26 well-imputed variables; given that fluid_input is the highest-ranked SHAP predictor, sensitivity analyses across complete-case, PMM, and missForest imputation strategies are presented in Additional file 1 (Supplementary Material 7, Table S5). Per-variable diagnostics are reported in Supplementary Material 2.

### Feature selection

The Elastic Net and Boruta methods were sequentially applied to identify relevant features from the training set. Six core features were confirmed by both methods: history of congestive heart failure, fluid intake within the first 24 h after ICU admission, SAPS-II score, SOFA score, vasopressor use, and CCI index. Subsequently, using Borda scores, two additional features were selected from the remaining features identified by both methods: GCS score and history of atrial fibrillation. The final 8 features were used for model construction (the top 10 features identified by Elastic Net and Boruta individually, as well as the Borda score integration process results, are presented in (Supplementary Material 3).

### Machine learning model development and optimal model selection

**Fig. 2:**
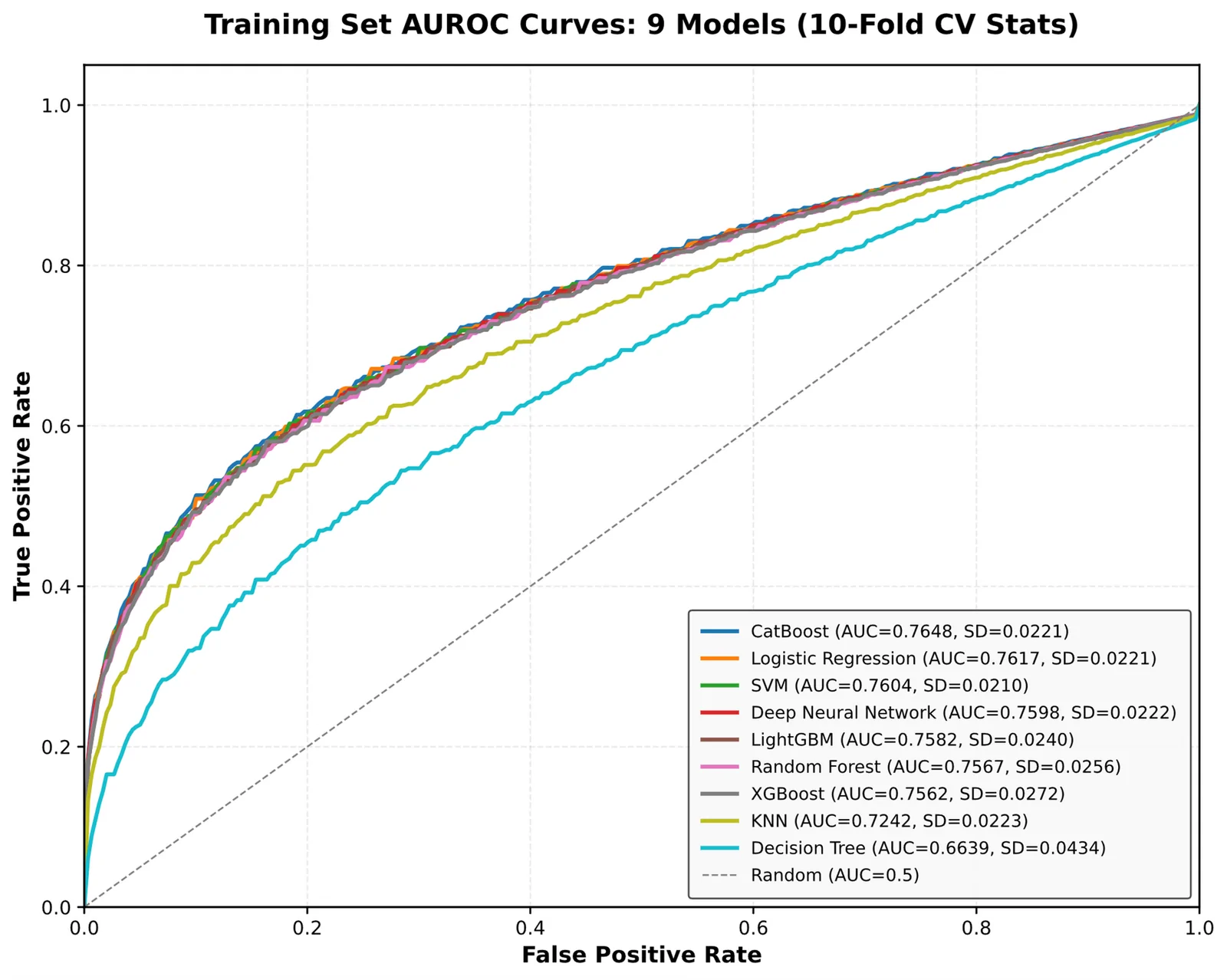
Summary of AUROC curves from the 10-fold cross-validation of the nine prediction models (Training Dataset)

Nine machine learning models were trained on the eight selected features. Their 10-fold cross-validation discrimination metrics (mean AUC, standard deviation, and 95% confidence interval) are reported in Table 1 and visualised in Fig. 2. CatBoost achieved the highest mean CV-10 AUC, followed closely by Logistic Regression; the magnitude of the difference between these two models was small relative to the spread across the nine algorithms as a whole. Pairwise statistical comparison between models is presented in the following paragraph (with multiplicity correction); the rationale for selecting CatBoost — informed by interpretability and deployment-node considerations rather than CV-AUC magnitude alone — is set out in the Discussion (“Selection of the Deployed Model”).

**Table 1:** Summary of performance metrics for the nine prediction models

Model	CV10 AUC	SD	CV 10 95%CI	Training Set AUC	Training Set 95% CI	Accuracy	F1 Score	Sensitivity	Specificity	PPV	NPV
CatBoost	0.7648	0.0221	0.7511–0.7786	0.784	0.761–0.808	0.775	0.526	0.421	0.921	0.697	0.792
LR	0.7617	0.0221	0.7480–0.7754	0.764	0.740–0.788	0.756	0.478	0.376	0.917	0.657	0.777
SVM	0.7604	0.0210	0.7474–0.7735	0.762	0.738–0.786	0.757	0.475	0.371	0.921	0.663	0.776
DNN	0.7598	0.0222	0.7460–0.7735	0.770	0.746–0.793	0.761	0.504	0.409	0.894	0.629	0.787
LightGBM	0.7582	0.0240	0.7433–0.7731	0.810	0.788–0.832	0.765	0.389	0.252	0.982	0.852	0.756
RF	0.7567	0.0256	0.7409–0.7726	0.886	0.868–0.904	0.832	0.645	0.514	0.963	0.850	0.818
XGBoost	0.7562	0.0272	0.7393–0.7730	0.782	0.759–0.805	0.750	0.576	0.573	0.773	0.542	0.833
KNN	0.7242	0.0223	0.7104–0.7380	0.832	0.811–0.853	0.786	0.562	0.461	0.924	0.718	0.802
DT	0.6639	0.0434	0.6370–0.6908	0.877	0.859–0.896	0.828	0.676	0.611	0.918	0.765	0.856

**Fig. 3:**
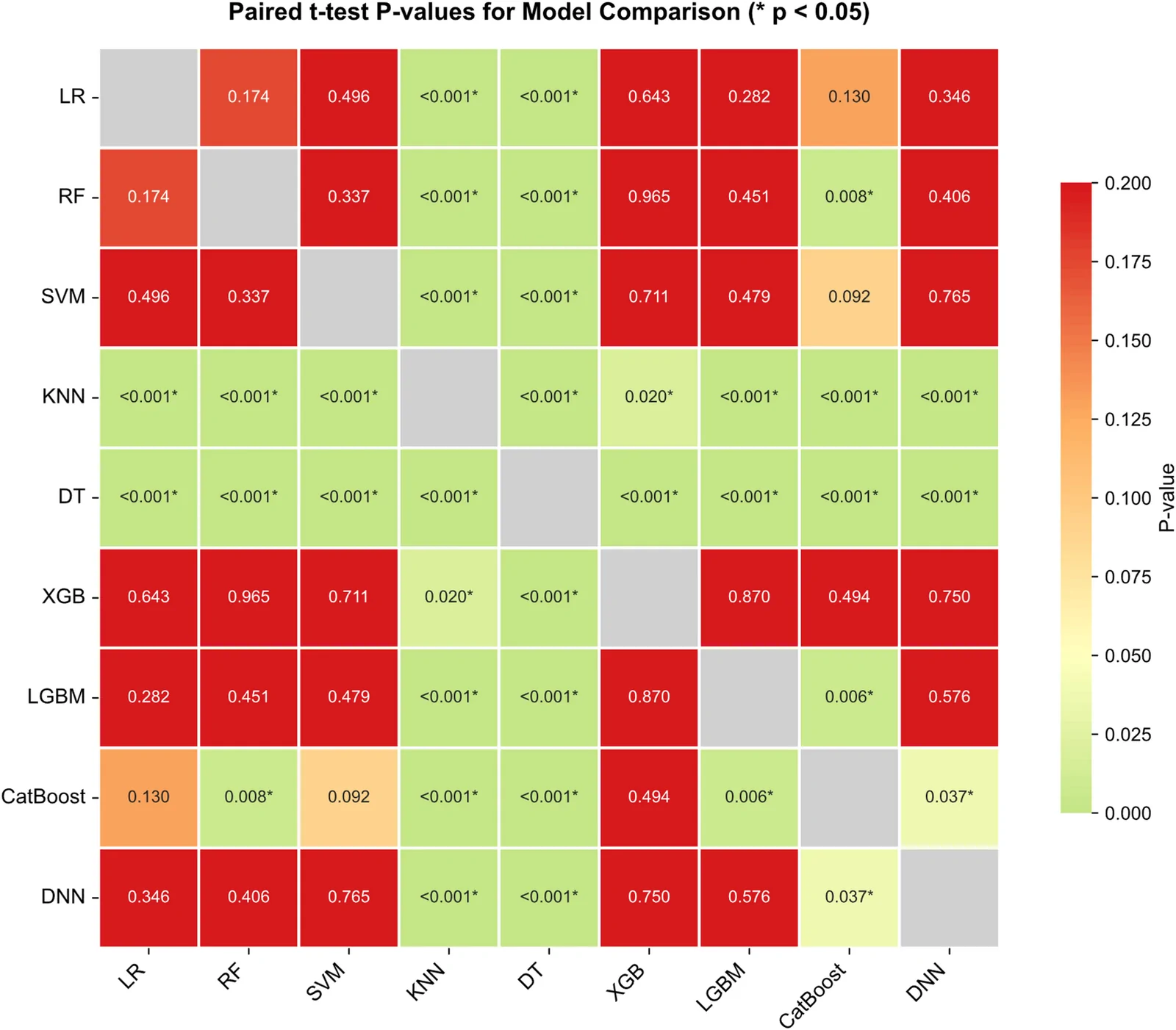
Heatmap of paired t-test comparisons among the nine prediction models. Left panel: uncorrected p-values (significance at *p* < 0.05). Right panel: Bonferroni-adjusted p-values (per-test threshold ≈ 0.0014 for 36 comparisons). The Benjamini–Hochberg FDR-adjusted q-values for the same 36 comparisons are tabulated in Supplementary Material 7 (Table S9)

Paired t-tests across the 36 model-pair comparisons identified 18 differences as significant under the uncorrected threshold (*p* < 0.05). Under Bonferroni correction (per-test threshold ≈ 0.0014), 14 contrasts remained significant; under Benjamini–Hochberg FDR (q = 0.05), 17 remained significant. Among contrasts directly relevant to model selection, CatBoost vs. Logistic Regression remained non-significant under all three procedures (uncorrected *p* = 0.1295; Bonf-*p* = 1.000; BH q = 0.233), while CatBoost vs. the weak baselines KNN and Decision Tree survived both corrections (Bonf-*p* ≤ 0.001; BH q ≤ 0.001). Once multiplicity is controlled, the apparent superiority of CatBoost over Logistic Regression and over the other top-tier models is not supported by the cross-validation evidence; the choice should be guided by parsimony and clinical interpretability rather than discrimination differences alone. The pairwise heatmaps are shown in Fig. 3 (left: uncorrected; right: Bonferroni-adjusted); the complete 36-row tables are provided in Supplementary Material 4 (Table S4, uncorrected) and Supplementary Material 7 (Table S9, multiplicity-corrected).

### Internal and external validation of the optimal model

**Fig. 4:**
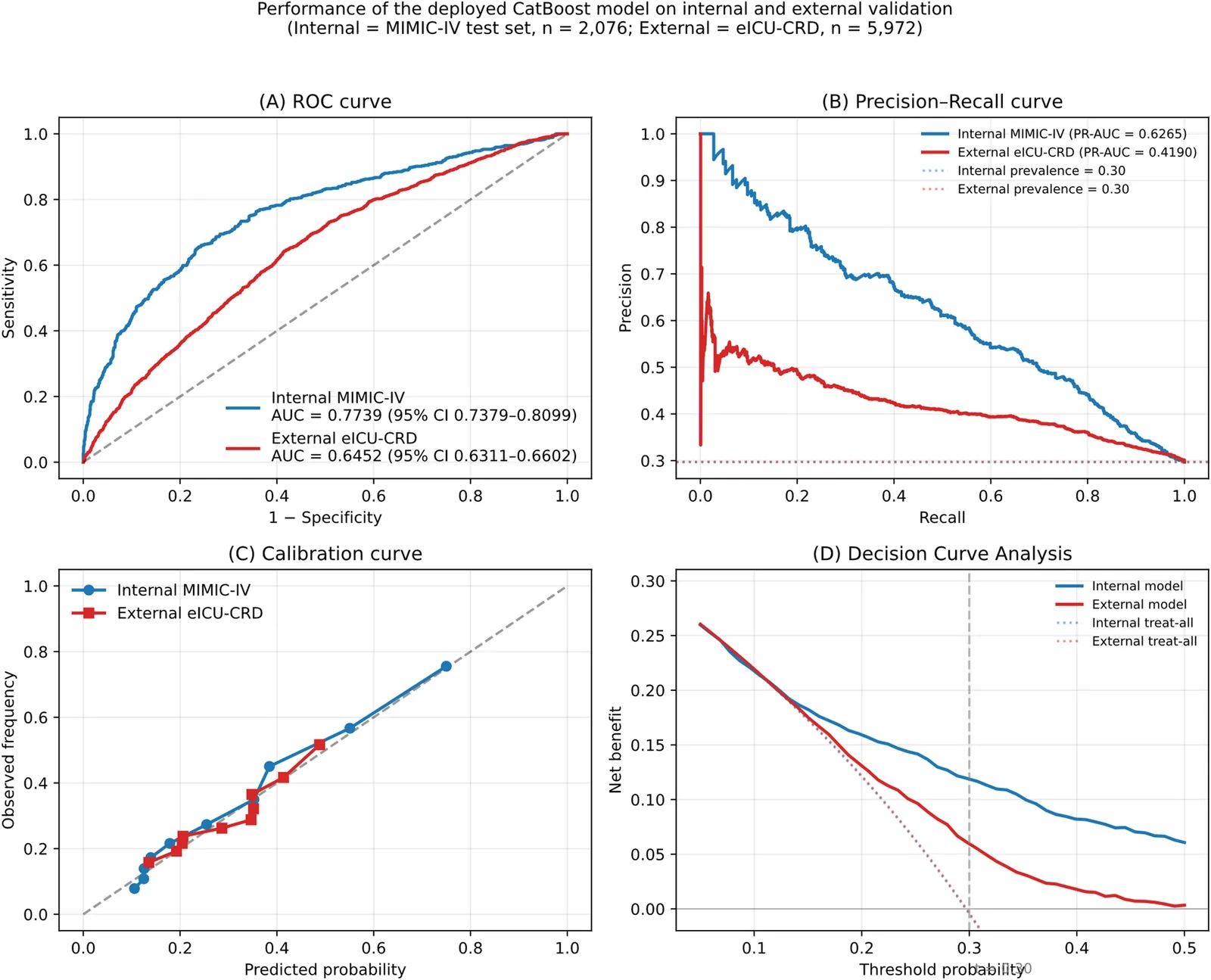
Performance of the deployed CatBoost model on internal (MIMIC-IV, *n* = 2,076) and external (eICU-CRD 2.0, *n* = 5,972) cohorts. (**A**) Receiver operating characteristic curve. (**B**) Precision–recall curve. (**C**) Calibration curve (10-bin quantile). (**D**) Decision curve analysis with the prevalence-anchored threshold of 0.30 marked. Internal cohort in blue, external cohort in red

Internal Validation: On the MIMIC-IV internal validation set (*n* = 2,076), the model achieved an AUC of 0.7739 (95% CI 0.7379–0.8099, Fig. 4A), PR-AUC 0.6265 (Fig. 4B), and Brier score 0.1628 (Fig. 4C). Calibration was satisfactory: the Hosmer–Lemeshow test did not reject good fit (χ² = 10.63, *p* = 0.224); calibration slope was 0.973 and intercept − 0.103, indicating that internal predictions are neither systematically over- nor under-confident; ICI was 0.036 and E/O ratio 1.048. Decision Curve Analysis (DCA) at the prevalence-anchored threshold range (t = 0.20 to 0.40) showed that the model yielded positive net benefit relative to both “treat all” and “treat none” strategies on the internal cohort, with NB at the prevalence-anchored operating point t = 0.30 of 0.061 (Fig. 4D); the operating-point characteristics at this threshold are reported below alongside the external-cohort analysis.

**Fig. 5:**
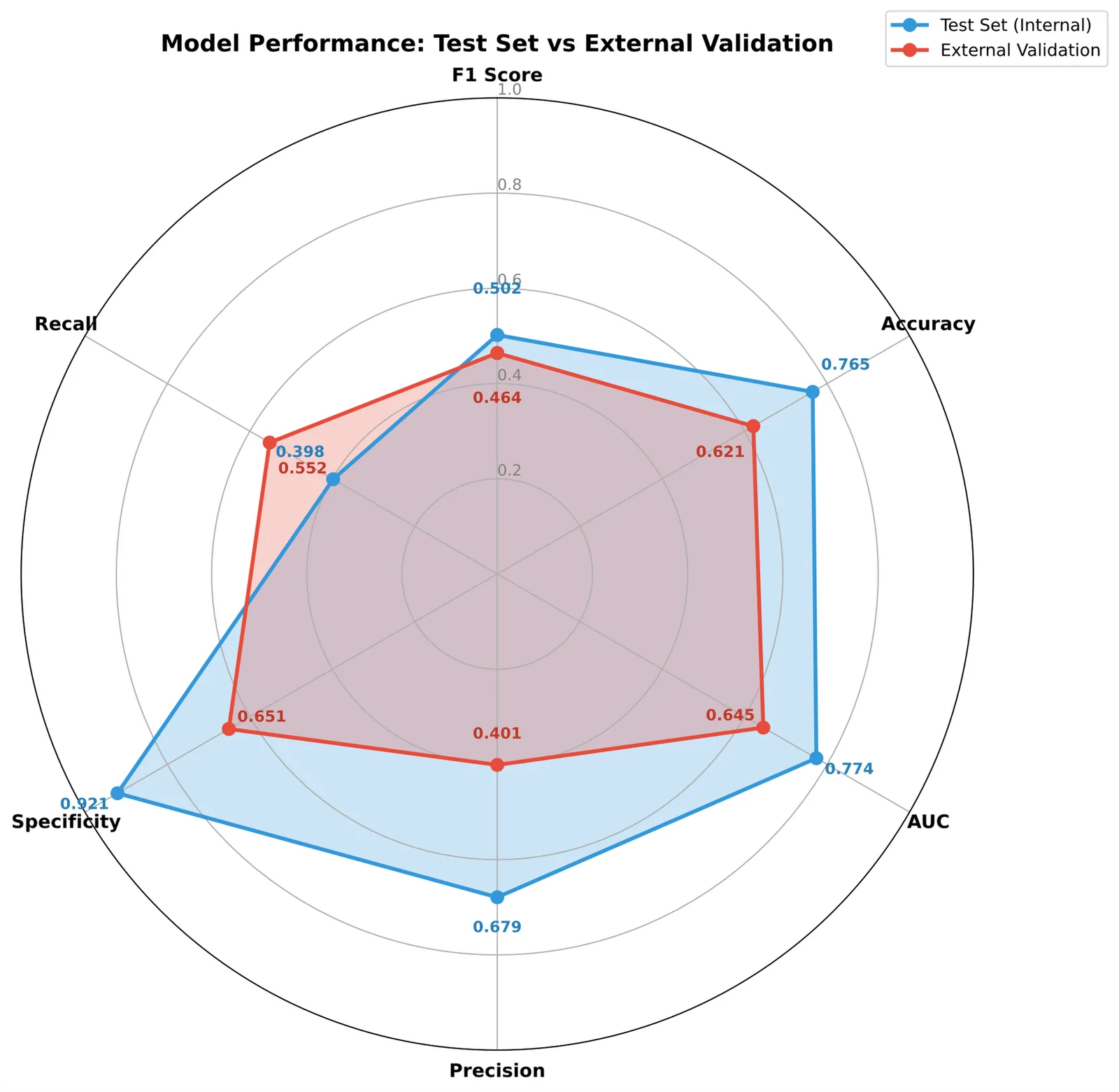
Performance comparison of the catboost model: internal vs. external validation

External Validation: On the eICU-CRD 2.0 external cohort (n = 5,972), the uncalibrated model achieved an AUC of 0.6452 (95% CI 0.6311–0.6602, Fig. 4A), PR-AUC 0.420 (Fig. 4B), and Brier score 0.202 (Fig. 4C). The calibration panel revealed substantial deterioration relative to the internal cohort (Hosmer-Lemeshow χ² = 121.73, p < 0.001; calibration slope 0.634; intercept − 0.341; ICI 0.049; E/O 1.047), reflecting systematic over-confidence on the external cohort. A 5-fold cross-validated logistic recalibration restored calibration to near-ideal (HL χ² 23.12, p = 0.003; slope 0.998; intercept − 0.002; ICI 0.023; E/O 1.000) without altering discrimination (rank order unchanged). Decision Curve Analysis at the prevalence-anchored threshold range (t = 0.20 to 0.40) showed positive net benefit relative to both ‘treat all’ and ‘treat none’ across the window, with NB = 0.059 at t = 0.30 (≈ outcome prevalence) where ‘treat all’ collapses to zero net benefit (full grid in Supplementary Material 7, Table S11). At the prevalence-anchored operating point t = 0.30, the model flagged 2,446 of 5,972 patients (41.0%), with sensitivity 0.55, specificity 0.65, PPV 0.40, NPV 0.77, and F1 0.46 (operating-point characteristics across thresholds 0.20–0.40 in Supplementary Material 7, Table S12). A multi-dimensional comparison is presented in the radar chart (Fig. 5). These results confirm cross-database generalizability with modest but clinically positive utility in the prevalence-anchored decision range.

### Model interpretability through SHAP analysis

**Fig. 6:**
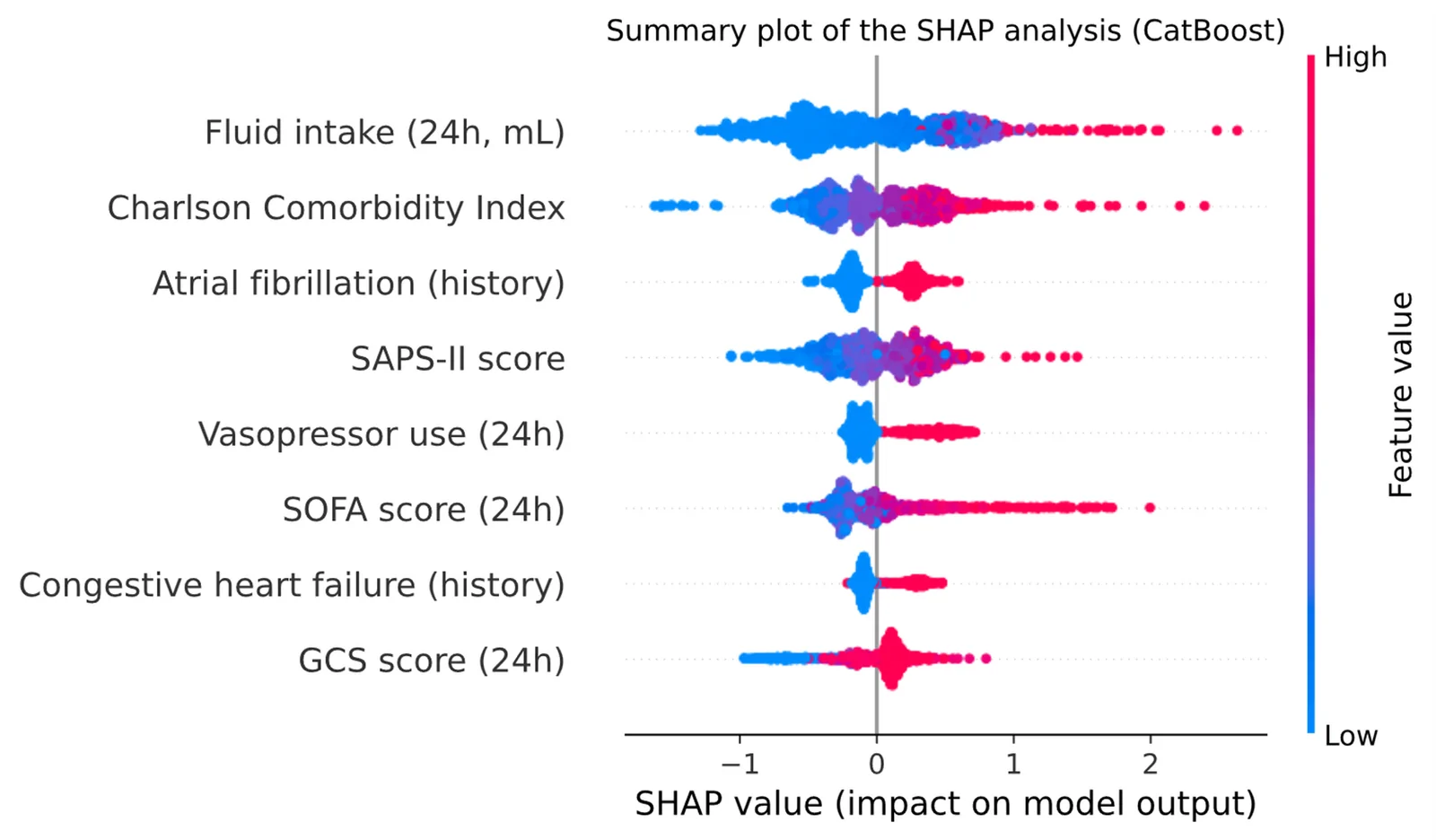
Summary plot of the SHAP analysis(CatBoost)

SHAP-based feature importance analysis was conducted to enhance the clinical interpretability of the optimal CatBoost model and overcome the “black box” limitation of machine learning algorithms. The SHAP summary plot (Fig. 6) illustrates the global feature importance rankings of the eight model inputs and their directional impact on model predictions. SHAP analysis revealed a ranked hierarchy of predictive factors: (1) fluid intake within the first 24 h after ICU admission—the most influential feature, with predominantly positive impact on prolonged ICU stay prediction; (2) Charlson Comorbidity Index—demonstrating consistent positive association with prolonged ICU LOS; and (3) history of atrial fibrillation—showing strong positive predictive impact. These three features were identified as the primary drivers of the model’s risk stratification.

SHAP analysis at the patient level, comprising individual waterfall plots from typical cases and dependence plots for the eight key features, further illustrates how specific feature values contribute to model predictions. These visualizations enable patient-level decision support and provide transparent, interpretable risk explanation aligned with clinical reasoning. (Supplementary Material 5)

### Development and implementation of interactive clinical decision support tool

An interactive web-based risk prediction calculator was developed and deployed at https://lupengyuchnhn.github.io/CABGLOSICU-Risk-Calculator-/ to translate the CatBoost model into a practical clinical decision support tool. This calculator accepts eight clinically-derived model inputs and generates risk probability estimates, enabling clinicians to rapidly assess the probability of prolonged ICU LOS for individual CABG patients within 24 h of ICU admission (website screenshot provided in Supplementary Material 6).

## Discussion

This study developed and validated a machine learning-based risk prediction model for prolonged postoperative ICU LOS in CABG patients using two large critical care databases (MIMIC-IV 3.1 for model development; eICU-CRD 2.0 for external validation). Through multi-dimensional feature selection from 55 perioperative candidate variables, eight key predictive features were identified: fluid intake within 24 h of ICU admission, Charlson Comorbidity Index (CCI), history of atrial fibrillation, Simplified Acute Physiology Score II (SAPS-II), Sequential Organ Failure Assessment (SOFA) score, vasopressor use, history of congestive heart failure, and Glasgow Coma Scale (GCS) score. Among nine evaluated machine learning algorithms, CatBoost achieved the highest cross-validation AUC; the difference relative to Logistic Regression was not statistically significant once multiple-testing correction was applied (see Section  “Machine learning model development and optimal model selection”), and CatBoost was retained on two grounds independent of discrimination magnitude: it captures non-linear and interaction effects without manually-specified terms, and supports a SHAP-based per-patient interpretability layer that anchors the deployed calculator (see Discussion subsection “Selection of the deployed model” for full rationale). SHAP analysis revealed that fluid intake within 24 h of ICU admission, CCI, and history of atrial fibrillation were the three most influential predictive factors for prolonged ICU LOS.

In summary, the eight key features incorporated in the predictive model are all clinically accessible indicators that are simple and practical to use. These features can be systematically categorized into two complementary domains: hemodynamic parameters (fluid intake, history of congestive heart failure, history of atrial fibrillation, vasopressor use) and multisystem organ function assessment scores (SAPS-II, SOFA, GCS, CCI). This dual-domain approach comprehensively represents the overall physiologic status and functional level of CABG patients postoperatively, with clear clinical predictive relevance.

### Hemodynamic domain

Fluid intake within the initial 24 h post-ICU admission emerged as the most influential predictive factor. This finding reflects the complex pathophysiology of cardiac surgery: increased capillary permeability at the surgical site, impaired myocardial contractility, and systemic inflammatory activation result in myocardial edema formation [10, 11]. Excessive fluid administration exacerbates myocardial edema, delays functional recovery, and increases both ICU LOS and mortality risk. This is supported by Bellos et al.‘s meta-analysis, which demonstrated that fluid overload in cardiac surgery patients is independently associated with prolonged mechanical ventilation and extended ICU LOS [12]. Patients with preexisting congestive heart failure or atrial fibrillation are particularly vulnerable to postoperative hemodynamic instability. Following surgical trauma and cardiopulmonary bypass, these patients frequently develop low cardiac output syndrome and systemic vasodilation, leading to circulatory compromise that significantly increases postoperative complications and prolonged ICU stay. Fudulu et al.‘s analysis documented that preoperative atrial fibrillation in CABG patients predicts multiple adverse outcomes including increased mortality, stroke risk, and need for renal replacement therapy, each contributing to prolonged ICU LOS [13]. Postoperative vasopressor administration, while necessary to maintain systemic perfusion, imposes considerable cardiac physiologic stress. Vasopressors increase both cardiac preload and afterload while elevating myocardial oxygen consumption, potentially compromising cardiac output and function. Damarlapally et al.‘s systematic review demonstrated that high Vasoactive-Inotropic Scores (VIS > 5) following CABG are significantly associated with multiple adverse outcomes including mortality and prolonged ICU LOS, supporting the mechanistic linkage between vasopressor burden and adverse perioperative trajectories [14].

### Multisystem organ function assessment domain

The SAPS-II scoring system objectively reflects the overall severity of patient illness and has been widely applied as a mature severity assessment tool for critically ill patients. Research by Xu et al. demonstrated that SAPS-II performs well in predicting ICU LOS in cardiac surgery patients [15]. In our study, SAPS-II scores in the prolonged ICU stay group were significantly higher than in the non-prolonged group, consistent with the above findings, and its predictive value was further verified. The SOFA score, as a scoring system for quantitatively assessing the degree of organ dysfunction in critically ill patients, has been widely applied in clinical practice in recent years [16]. Research demonstrates that SOFA score is an independent predictor of early stroke following CABG, and SOFA score is directly related to adverse patient outcomes [17]. In single-sample SHAP analysis, SOFA scores in the prolonged ICU stay group were significantly higher than in the non-prolonged group, further confirming the value of SOFA score in assessing overall disease severity and organ function status. The Charlson Comorbidity Index (CCI), proposed in 1987, has been widely applied in clinical practice to date [18]. Research demonstrates that CCI can serve as an effective tool for five-year mortality risk stratification in CABG patients (HR 1.38 [1.33–1.43], *p* < 0.001), as well as predicting ICU LOS [19]. In single-sample SHAP analysis, CCI in the prolonged ICU stay group was substantially higher than in the non-prolonged group, further confirming the importance of CCI in prognostic assessment of CABG patients. From the SHAP visualization analysis of the optimal prediction model, the relationship between GCS and risk of prolonged ICU stay is predominantly protective in nature—that is, patients with better postoperative neurological function tend to have better outcomes, which is readily understandable. However, in this model, GCS has limited impact, as its importance ranks lowest among the eight included features.

### Model performance and clinical applicability

Internal MIMIC-IV AUC was 0.7739 (95% CI 0.7379–0.8099) with adequate calibration (slope 0.973; Hosmer–Lemeshow *p* = 0.224); external eICU-CRD 2.0 uncalibrated AUC was 0.6452 (95% CI 0.6311–0.6602), with calibration recoverable through a simple intercept-and-slope update (slope 0.998) rather than full retraining. This performance band is comparable to the C-index of 0.653–0.793 reported for machine-learning variants of the Society of Thoracic Surgeons risk model on softer endpoints [20], although our model addresses a distinct clinical question — prolonged ICU LOS at the 24-hour evaluation moment rather than peri-operative mortality.

### Selection of the deployed model

Cross-validation discrimination between CatBoost and Logistic Regression is statistically equivalent under multiple-testing control (uncorrected *p* = 0.1295; Bonferroni 1.000; BH q = 0.233; Section “Machine learning model development and optimal model selection”); we therefore do not claim discrimination superiority. CatBoost was retained on two non-discrimination grounds: it captures non-linear and interaction effects among the eight features without manually-specified terms (Fig. 6), and supports SHAP-based per-patient interpretability under tree-ensemble Shapley estimation. Variance Inflation Factor analysis confirmed that overlap among severity scores did not reach problematic multicollinearity (range 1.07–3.17), with reduced models on either block alone under-performing the eight-feature specification (Supplementary Material 7, Tables S14a–S14b). A parsimony-preferring Logistic Regression sibling (CV-AUC 0.7632 ± 0.0155; internal test 0.7356; external uncalibrated 0.6227) is released in Supplementary Material 7 (Table S10) for users preferring a directly-inspectable linear model.

### Comparison with preoperative reference model

A preoperative-only logistic-regression reference (5-fold cross-validated) was fitted on the eICU cohort using age, sex, CCI, congestive heart failure and atrial fibrillation history, and cardiac ICU admission — variables analogous to those in three published preoperative risk-prediction models [21–23]. It yielded AUC 0.659 (95% CI 0.644–0.673), slope 0.976, ICI 0.020, against the 24-hour CatBoost’s AUC 0.6452 (95% CI 0.6311–0.6602), slope 0.998, ICI 0.023 after recalibration. Decision-curve analysis showed no incremental net benefit (ΔNB − 0.010 to ≈ 0.000 across t = 0.20–0.45); we therefore claim neither discrimination nor net-benefit superiority. The 24-hour model’s contribution lies elsewhere: a distinct deployment moment (post-operative 24-hour, after preoperative inputs have informed earlier decisions); contemporaneous post-operative covariates (24-hour fluid intake, SOFA, SAPS-II) that materially shift individual-level risk attribution; and bedside SHAP-based per-patient attribution. It also adds independent eICU-CRD 2.0 external validation and a parsimony-preferring Logistic Regression sibling, complementing a directly-comparable postoperative MIMIC-IV ICU-LOS prediction model [24].

### Study limitations

Cohort derivation from public databases introduces residual selection bias and inter-regional variation; intraoperative hemodynamic and surgical-complexity variables are absent; and age, sex, BMI, and race were dropped by Elastic Net + Boruta selection. The most influential SHAP predictor (24-hour fluid intake) shows the largest median imputation bias (13.76%; KS *p* = 0.0002), and discrimination is modest at the prevalence-anchored t = 0.30 (sensitivity 0.55, specificity 0.65, PPV 0.40, NPV 0.77, F1 0.46); SHAP-based attributions therefore complement rather than replace binary classification, with conclusions backed by complete-case, predictive mean matching, and missForest sensitivity analyses (Supplementary Material 7, Tables S5–S6). Eligibility retained 324 short-stay patients (< 24 h, 4.7%); refitting without them left the SHAP top-3 unchanged. The > 3-day cutpoint reflects clinical convention, deployment-node decision context, and prevalence balance, with a > 5-day sensitivity analysis preserving the qualitative SHAP ranks. The 24-hour fluid signal accumulates in ICU hours 6–24, raising a reverse-causation concern not fully resolvable without prospective causal labelling. Finally, both cohorts originate from US academic centres (applicability to Thailand and other Asian centres unverified), dynamic updating is absent, and substantial uncalibrated eICU drift (slope 0.634) was corrected by logistic recalibration (0.998); non-US deployment should mirror this local recalibration step before prospective clinical use.

### Future directions

Future work should incorporate dynamic variables from intraoperative and ICU treatment phases (e.g., continuous intraoperative hemodynamic parameters) to develop dynamic prediction models capable of real-time risk assessment updates, thereby improving prediction timeliness.

## Conclusion

This study developed a decision-support tool at the post-CABG ICU 24-hour evaluation moment using interpretable machine learning and eight readily-accessible clinical features obtained within the first 24 h after ICU admission: 24-hour fluid intake, CCI, history of atrial fibrillation, SAPS-II score, vasopressor use, SOFA score, history of congestive heart failure, and GCS score. Among the nine candidate algorithms evaluated, CatBoost achieved the highest cross-validation AUC; the difference relative to Logistic Regression was not statistically significant once multiple-testing correction was applied (see Section  “Machine learning model development and optimal model selection”), and CatBoost was retained as the deployed model on grounds of non-linear interaction capture and SHAP-based per-patient interpretability rather than discrimination superiority. The eight identified key predictive factors possess clear clinical significance and physiologic basis, are simple and practical to use, and provide valuable guidance for clinical practice. The online risk calculator developed based on the CatBoost model supports rapid bedside risk assessment for individual CABG patients at the 24-hour ICU evaluation moment, complementing established preoperative risk stratification.

## Supplementary Information

Below is the link to the electronic supplementary material.

Supplementary Material 1

## Data Availability

The datasets analysed in this study are publicly available. MIMIC-IV 3.1 can be accessed through https://mimic.mit.edu/, and eICU-CRD 2.0 can be accessed through https://eicu-crd.mit.edu/. Prior to access, registration on the respective platform and completion of necessary data usage training are required according to data privacy regulations. The author Pengyu Lu has completed the CITI exam (certificate code: 13605560). All code, analysis scripts, intermediate cohort data, result-summary JSONs, and supporting documentation (TRIPOD+AI checklist, data dictionary, reproduction README) are archived at Zenodo with a permanent DOI (https://doi.org/10.5281/zenodo.20135180). In addition to the primary CatBoost calculator deployed at https://lupengyuchnhn.github.io/CABGLOSICU-Risk-Calculator-/, a parsimony-preferring Logistic Regression secondary calculator is released; its full coefficient table and scaler parameters are provided in Supplementary Material 7 (Table S10), and patient-level external predictions on the eICU cohort together with a usage README are archived alongside the primary CatBoost model on Zenodo.

## Abbreviations

AUC: Area Under the Receiver Operating Characteristic Curve
AUROC: Area Under the Receiver Operating Characteristic Curve
AUPRC: Area Under the Precision-Recall Curve
BH-FDR: Benjamini-Hochberg False Discovery Rate
CABG: Coronary Artery Bypass Grafting
CatBoost: Categorical Boosting
CCI: Charlson Comorbidity Index
CI: Confidence Interval
CITI: Collaborative Institutional Training Initiative
CV: Cross-Validation
DCA: Decision Curve Analysis
DNN: Deep Neural Network
DOI: Digital Object Identifier
DT: Decision Tree
E/O: Expected-to-Observed ratio
eICU-CRD: eICU Collaborative Research Database
F1: F1-score (harmonic mean of precision and recall)
FDR: False Discovery Rate
GCS: Glasgow Coma Scale
HIPAA: Health Insurance Portability and Accountability Act
HL: Hosmer-Lemeshow
ICI: Integrated Calibration Index
ICMJE: International Committee of Medical Journal Editors
ICU: Intensive Care Unit
IQGI: Imputation Quality Global Index
IQR: Interquartile Range
KNN: K-Nearest Neighbors
KS: Kolmogorov-Smirnov
LightGBM: Light Gradient Boosting Machine
LOS: Length of Stay
LR: Logistic Regression
MICE: Multivariate Imputation by Chained Equations
MIMIC-IV: Medical Information Mart for Intensive Care IV
ML: Machine Learning
NB: Net Benefit
NPV: Negative Predictive Value
OASIS: Oxford Acute Severity of Illness Score
PPV: Positive Predictive Value
PR: Precision-Recall
PR-AUC: Precision-Recall Area Under the Curve
RF: Random Forest
ROC: Receiver Operating Characteristic
SAPS-II: Simplified Acute Physiology Score II
SHAP: SHapley Additive exPlanations
SOFA: Sequential Organ Failure Assessment
SQL: Structured Query Language
TRIPOD + AI: Transparent Reporting of a multivariable prediction model for Individual Prognosis Or Diagnosis + Artificial Intelligence extension
VIF: Variance Inflation Factor
XGBoost: Extreme Gradient Boosting
