# Supplementary material for: Development and external validation of an interpretable machine learning model for predicting prolonged postoperative ICU length of stay in coronary artery bypass grafting patients using MIMIC-IV 3.1 and eICU-CRD 2.0
Source: BMC Med Inform Decis Mak. 2026 Jun 27;26:353. doi: 10.1186/s12911-026-03659-y (PMC13579721; doi:10.1186/s12911-026-03659-y)
Supplement: Supplementary file 1 [file 12911_2026_3659_MOESM1_ESM.docx]

**Supplementary Materials**

**Supplementary Material 1** Baseline Characteristics of the Study Population

Table S1. Baseline Characteristics of the Study Population

**Supplementary Material 2.** Data Imputation Quality Assessment

Imputation quality was assessed at the per-variable level using the Kolmogorov–Smirnov (KS) two-sample distributional consistency test for continuous variables and chi-square distribution testing for categorical variables. Aggregate composite scores are reported for completeness — composite score for continuous variables 94.66 (mean preservation 99.53, variance preservation 98.05, distribution consistency 88.46%), composite score for categorical variables 99.89 (distribution preservation 99.78%, chi-square consistency 100.00%) — but should not be read as a per-variable certificate. Of the 29 variables requiring imputation, 26 satisfied KS distributional consistency (p > 0.05). Three variables did not pass and merit explicit disclosure: fluid intake within the first 24 hours of ICU admission (12.08 % missing; KS p = 0.0002; median bias 13.76 %); temperature (10.72 % missing; KS p = 0.0011; median bias 0.11 %); and invasive ventilation hours (22.07 % missing; KS p < 0.001; median bias 25.17 %). Because fluid intake is the highest-ranked SHAP feature in our model, this imputation limitation is material and is documented in the Limitations subsection "Imputation fidelity for fluid_input" of the main manuscript. To verify the robustness of our principal findings to imputation choice, sensitivity analyses were performed under three alternative strategies (complete-case, predictive mean matching with m = 5, and missForest random-forest iterative imputation); the dominant SHAP predictor (fluid intake) and the second-ranked predictor (Charlson Comorbidity Index) preserved identical rank order across all three strategies, and the external eICU-CRD 2.0 area under the curve ranged from 0.5927 to 0.6097 across strategies, consistent with imputation-induced variability of approximately three to five percentage points relative to the primary external AUC of 0.6452. Per-variable KS diagnostics are provided in Table S2 and Figure S1; the three-strategy sensitivity analysis is provided in Additional file 1, Table S5. Detailed imputation-quality data are provided below:

Table S2. Details of MICE Imputation for Continuous Features.

Figure S1. Distribution Comparison Plots for MICE Imputation of Continuous Features.

Table S3. Details of Categorical Feature Imputation.

Figure S2. Comparison Plots for Categorical Feature Imputation.

**Supplementary Material 3.** Top 10 Features Identified by Elastic Net and Boruta, and Borda Count Integration Process

Using the Elastic Net method, the top 10 ranked features were: Charlson Comorbidity Index (CCI), history of Congestive Heart Failure, history of Atrial Fibrillation, SOFA score, SAPS-II score, OASIS score, GCS score, Fluid Input within the first 24 hours of ICU admission, peripheral oxygen saturation (SpO₂), and use of Vasoactive Drugs.

Using the Boruta method, the feature selection state evolution and importance convergence plot are shown in Figure S3. The top 10 ranked features were: Fluid Input within the first 24 hours of ICU admission, CCI, Total Urine Output within the first 24 hours of ICU admission, SAPS-II score, SOFA score, Blood Urea Nitrogen (BUN), use of Vasoactive Drugs, Creatinine, Age, and history of Congestive Heart Failure.

A core set of six features was consistently confirmed by both methods: history of Congestive Heart Failure, Fluid Input within the first 24 hours of ICU admission, SAPS-II score, SOFA score, use of Vasoactive Drugs, and CCI. Subsequently, two additional features were integrated based on their Borda scores from the remaining features identified by each method: GCS score and history of Atrial Fibrillation. These eight features were used to construct the prediction model.

Figure S3. Coefficient Path Plot for the Elastic Net Regression.

Figure S4. Feature Selection State Evolution and Importance Convergence Plot for the Boruta Algorithm.

Figure S5. Flowchart of the Dual Feature Selection Strategy (Elastic Net and Boruta) Integrated via Borda Count.

**Supplementary Material 4.** Detailed Paired t-Test Results for the Nine Prediction Models

Table S4. Detailed Results of Paired t-Test Comparisons Among the Nine Models (uncorrected p-values, with α = 0.05). Multiplicity-corrected results — including Bonferroni-adjusted p-values and Benjamini–Hochberg FDR-adjusted q-values — are reported in Table S9.

**Supplementary Material 5.** SHAP analysis Two Representative Case Studies and eight feature Dependence plots

Two representative case studies with individual SHAP waterfall plots demonstrating how the eight features contribute to risk predictions for a low-risk and high-risk patient, respectively

SHAP dependence plots for each of the eight features, revealing the relationship between feature values and model predictions, along with potential feature interactions

Figure S6. A Low-Risk Patient with Non-Prolonged ICU Stay (Lowest Predicted Probability: 0.057).

Figure S7. A High-Risk Patient with Prolonged ICU Stay (Highest Predicted Probability: 0.945).

Figure S8. SHAP Dependence Plot - Fluid Input within First 24 Hours of ICU Admission

Figure S9. SHAP Dependence Plot - Charlson Comorbidity Index (CCI)

Figure S10. SHAP Dependence Plot - History of Atrial Fibrillation

Figure S11. SHAP Dependence Plot - Simplified Acute Physiology Score II (SAPS-II)

Figure S12. SHAP Dependence Plot - SOFA Score

Figure S13. SHAP Dependence Plot - Vasopressor Use

Figure S14. SHAP Dependence Plot - History of Congestive Heart Failure

Figure S15. SHAP Dependence Plot - Glasgow Coma Scale (GCS) Score

**Supplementary Material 6.** Development of the Online Risk Prediction Calculator

Figure S16. Screenshot of the Online Risk Prediction Calculator.

Table S1. Baseline Characteristics of the Study Population

|  | **MIMICIV3.1(n=6919)** | | | | **eICU-CRD2.0(n=5972)** | | |
| --- | --- | --- | --- | --- | --- | --- | --- |
| **Feature** | | **Non-Prolonged ICU Stay Group (n=4865)** | **Prolonged ICU Stay Group (n=2054)** | **P-value** | **Non-Prolonged ICU Stay Group (n=4196)** | **Prolonged ICU Stay Group (n=1776)** | **P-value** |
| **Demographics** | |  |  |  |  |  |  |
| Age (years) | | 67.00 (60.00-73.00) | 70.00 (62.00-77.00) | ＜0.001 | 66.00 (59.00-73.00) | 68.00 (61.00-75.00) | <0.001 |
| Gender | | 4865 (100.0%) | 2054 (100.0%) | ＜0.001 | 4196 (100.0%) | 1776 (100.0%) |  |
| Female | | 945 (19.4%) | 585 (28.5%) |  | 980 (23.4%) | 550 (31.0%) |  |
| Male | | 3920 (80.6%) | 1469 (71.5%) |  | 3216 (76.6%) | 1226 (69.0%) | <0.001 |
| Race | | 4865 (100.0%) | 2054 (100.0%) | 0.020 | 4196 (100.0%) | 1776 (100.0%) |  |
| White | | 3400 (69.9%) | 1457 (70.9%) |  | 0 (0.0%) | 0 (0.0%) |  |
| Black | | 162 (3.3%) | 98 (4.8%) |  | 0 (0.0%) | 0 (0.0%) |  |
| Hispanic | | 149 (3.1%) | 59 (2.9%) |  | 135 (3.2%) | 43 (2.4%) |  |
| Asian | | 121 (2.5%) | 45 (2.2%) |  | 104 (2.5%) | 33 (1.9%) |  |
| Other | | 1033 (21.2%) | 395 (19.2%) |  | 3957 (94.3%) | 1700 (95.7%) |  |
| Height (cm) | | 173.00 (168.00-178.00) | 173.00 (163.00-178.00) | ＜0.001 | 173.00 (167.00-180.30) | 172.70 (163.00-178.00) | <0.001 |
| Weight (kg) | | 87.50 (76.80-99.70) | 85.55 (74.10-99.80) | 0.002 | 88.00 (75.90-100.70) | 86.61 (74.10-102.00) | 0.188 |
| BMI （Kg/m²） | | 29.32 (26.22-33.03) | 29.20 (26.06-33.42) | 0.813 | 29.17 (25.97-33.19) | 29.49 (25.63-34.14) | 0.111 |
| Emergency Admission | | 4865 (100.0%) | 2054 (100.0%) | ＜0.001 | 4196 (100.0%) | 1776 (100.0%) |  |
| No | | 4332 (89.0%) | 1754 (85.4%) |  | 4178 (99.6%) | 1756 (98.9%) |  |
| Yes | | 533 (11.0%) | 300 (14.6%) |  | 18 (0.4%) | 20 (1.1%) | 0.004 |
| Insurance Type | | 4839 (99.5%) | 2035 (99.1%) | ＜0.001 | 4196 (100.0%) | 1776 (100.0%) |  |
| Medicare | | 2571 (52.8%) | 1375 (66.9%) |  | 2398 (57.1%) | 1031 (58.1%) |  |
| Private | | 1751 (36.0%) | 474 (23.1%) |  | 1361 (32.4%) | 556 (31.3%) |  |
| Medicaid | | 424 (8.7%) | 154 (7.5%) |  | 372 (8.9%) | 158 (8.9%) |  |
| Other | | 93 (1.9%) | 32 (1.6%) |  | 65 (1.5%) | 31 (1.7%) |  |
| Admission Season | | 4865 (100.0%) | 2054 (100.0%) | 0.810 | 4196 (100.0%) | 1776 (100.0%) |  |
| Spring | | 1246 (25.6%) | 505 (24.6%) |  | 994 (23.7%) | 458 (25.8%) |  |
| Summer | | 1224 (25.2%) | 516 (25.1%) |  | 1089 (26.0%) | 461 (26.0%) |  |
| Autumn | | 1223 (25.1%) | 524 (25.5%) |  | 1103 (26.3%) | 400 (22.5%) |  |
| Winter | | 1172 (24.1%) | 509 (24.8%) |  | 1010 (24.1%) | 457 (25.7%) |  |
| **Vital Signs** | |  |  |  |  |  |  |
| HR(beats/min) | | 80.00 (74.00-85.00) | 80.00 (76.00-88.00) | ＜0.001 | 82.93 (76.16-90.75) | 85.00 (78.11-91.92) | <0.001 |
| Sbp（mmHg） | | 112.00 (102.00-124.00) | 111.00 (99.00-125.00) | 0.023 | 115.71 (108.24-123.79) | 114.09 (106.83-122.79) | <0.001 |
| Dbp（mmHg） | | 59.00 (52.00-65.25) | 57.00 (50.00-66.00) | ＜0.001 | 56.43 (51.84-61.33) | 54.76 (49.49-59.55) | <0.001 |
| Mbp（mmHg） | | 77.00 (69.00-85.00) | 75.00 (67.00-85.00) | ＜0.001 | 74.95 (70.58-79.90) | 73.34 (68.57-78.51) | <0.001 |
| RR(beats/min) | | 15.50 (14.00-17.00) | 16.00 (14.00-18.00) | ＜0.001 | 17.51 (15.58-20.00) | 17.54 (15.39-20.09) | 0.790 |
| T（℃） | | 36.44 (36.10-36.67) | 36.44 (35.90-36.72) | 0.568 | 37.08 (36.79-37.38) | 37.12 (36.81-37.46) | <0.001 |
| Spo2（%） | | 100.00 (99.00-100.00) | 100.00 (98.00-100.00) | ＜0.001 | 97.51 (96.23-98.62) | 97.47 (96.10-98.77) | 0.828 |
| **Severity of Illness Scores** | |  |  |  |  |  |  |
| CCI | | 4.00 (3.00-5.00) | 5.00 (4.00-7.00) | ＜0.001 | 3.00 (2.00-5.00) | 4.00 (3.00-6.00) | <0.001 |
| SOFA | | 4.00 (3.00-6.00) | 6.00 (4.00-8.00) | ＜0.001 | 5.00 (4.00-8.00) | 7.00 (5.00-9.00) | <0.001 |
| SAPS-Ⅱ | | 35.00 (29.00-41.00) | 40.00 (34.00-48.00) | ＜0.001 | 39.00 (31.00-47.00) | 42.00 (35.00-52.00) | <0.001 |
| OASIS | | 31.00 (27.00-36.00) | 34.00 (29.00-39.00) | ＜0.001 | 23.00 (18.00-29.00) | 24.00 (20.00-31.00) | <0.001 |
| GCS | | 15.00 (14.00-15.00) | 15.00 (14.00-15.00) | 0.044 | 15.00 (10.00-15.00) | 15.00 (8.00-15.00) | 0.009 |
| **Laboratory Parameters** | |  |  |  |  |  |  |
| Cr（Umol/L） | | 0.90 (0.70-1.00) | 1.00 (0.80-1.30) | ＜0.001 | 0.96 (0.80-1.20) | 1.12 (0.90-1.50) | <0.001 |
| Bun（mg/dL） | | 15.00 (12.00-19.00) | 18.00 (14.00-25.00) | ＜0.001 | 16.00 (13.00-21.00) | 19.00 (15.00-26.00) | <0.001 |
| Na+（mmol/L） | | 138.00 (137.00-140.00) | 139.00 (137.00-141.00) | 0.058 | 141.00 (139.00-143.00) | 142.00 (140.00-144.00) | <0.001 |
| K+ （mmol/L） | | 4.30 (4.00-4.60) | 4.30 (4.00-4.70) | 0.083 | 4.50 (4.20-4.80) | 4.50 (4.30-4.80) | <0.001 |
| Glu（mmol/L） | | 6.49 (5.77-7.38) | 6.72 (5.77-7.94) | ＜0.001 | 169.00 (150.00-192.00) | 178.00 (156.00-205.00) | <0.001 |
| HB（g/dL） | | 9.70 (8.60-10.90) | 9.10 (7.90-10.50) | ＜0.001 | 11.10 (9.90-12.60) | 11.20 (9.90-13.38) | 0.004 |
| Plt（×10⁹/L） | | 146.00 (118.00-181.00) | 146.00 (114.00-192.00) | 0.304 | 154.00 (125.75-190.00) | 150.00 (120.00-190.00) | 0.005 |
| WBC（×10⁹/L） | | 12.10 (9.20-15.60) | 12.50 (9.15-16.40) | 0.006 | 13.93 (11.10-17.60) | 14.80 (11.50-19.00) | <0.001 |
| INR（sec） | | 1.40 (1.30-1.50) | 1.40 (1.30-1.60) | ＜0.001 | 1.30 (1.20-1.40) | 1.30 (1.20-1.50) | <0.001 |
| PTT（sec） | | 29.90 (27.00-34.20) | 32.00 (28.40-39.00) | ＜0.001 | 31.30 (28.00-35.40) | 33.00 (29.00-39.00) | <0.001 |
| Lac（mmol/L） | | 2.00 (1.50-2.60) | 2.10 (1.50-2.90) | ＜0.001 | 1.87 (1.31-2.66) | 2.50 (1.60-4.88) | <0.001 |
| Ph | | 7.40 (7.36-7.43) | 7.39 (7.35-7.43) | ＜0.001 | 7.39 (7.36-7.42) | 7.41 (7.37-7.44) | <0.001 |
| BUN-to-Creatinine Ratio (%) | | 18.33 (15.00-21.67) | 18.33 (15.00-22.50) | 0.278 | 16.49 (13.70-19.82) | 16.09 (12.90-19.78) | 0.001 |
| **Fluid Balance within 24h** | |  |  |  |  |  |  |
| Fluid-input（ml） | | 198.43 (89.74-567.57) | 639.57 (191.64-1686.37) | ＜0.001 | 925.94 (500.00-3018.39) | 1420.69 (500.00-3643.06) | <0.001 |
| Total-urine-output（ml） | | 1835.00 (1396.25-2415.00) | 1509.00 (1040.00-2125.00) | ＜0.001 | 900.00 (0.00-1738.50) | 618.00 (0.00-1887.50) | 0.726 |
| **Invasive Mechanical Ventilation Duration (h)** | | 24.00 (8.00-24.00) | 24.00 (12.08-24.00) | ＜0.001 | 4.00 (4.00-8.00) | 4.00 (4.00-12.00) | <0.001 |
| **Number of Bypass Grafts** | |  |  |  |  |  |  |
| Single Vessel | | 4865 (100.0%) | 2054 (100.0%) | 0.030 | 4196 (100.0%) | 1776 (100.0%) |  |
| No | | 3207 (65.9%) | 1410 (68.6%) |  | 4196 (100.0%) | 1775 (99.9%) |  |
| Yes | | 1658 (34.1%) | 644 (31.4%) |  | 0 (0.0%) | 1 (0.1%) | 0.297 |
| Two Vessels | | 4865 (100.0%) | 2054 (100.0%) | 0.033 | 4196 (100.0%) | 1776 (100.0%) |  |
| No | | 4103 (84.3%) | 1689 (82.2%) |  | 4196 (100.0%) | 1776 (100.0%) |  |
| Yes | | 762 (15.7%) | 365 (17.8%) |  | 0 (0.0%) | 0 (0.0%) | 1.000 |
| Three Vessels | | 4865 (100.0%) | 2054 (100.0%) | 0.058 | 4196 (100.0%) | 1776 (100.0%) |  |
| No | | 4384 (90.1%) | 1819 (88.6%) |  | 4196 (100.0%) | 1776 (100.0%) |  |
| Yes | | 481 (9.9%) | 235 (11.4%) |  | 0 (0.0%) | 0 (0.0%) | 1.000 |
| Four Vessels | | 4865 (100.0%) | 2054 (100.0%) | 0.866 | 4196 (100.0%) | 1776 (100.0%) |  |
| No | | 4617 (94.9%) | 1952 (95.0%) |  | 4196 (100.0%) | 1776 (100.0%) |  |
| Yes | | 248 (5.1%) | 102 (5.0%) |  | 0 (0.0%) | 0 (0.0%) | 1.000 |
| **Comorbidities** | |  |  |  |  |  |  |
| Myocardial Infarction | | 4865 (100.0%) | 2054 (100.0%) | ＜0.001 | 4196 (100.0%) | 1776 (100.0%) |  |
| No | | 2964 (60.9%) | 1067 (51.9%) |  | 2157 (51.4%) | 676 (38.1%) |  |
| Yes | | 1901 (39.1%) | 987 (48.1%) |  | 2039 (48.6%) | 1100 (61.9%) | <0.001 |
| Congestive Heart Failure | | 4865 (100.0%) | 2054 (100.0%) | ＜0.001 | 4196 (100.0%) | 1776 (100.0%) |  |
| No | | 3951 (81.2%) | 1174 (57.2%) |  | 3724 (88.8%) | 1398 (78.7%) |  |
| Yes | | 914 (18.8%) | 880 (42.8%) |  | 472 (11.2%) | 378 (21.3%) | <0.001 |
| Peripheral Vascular Disease | | 4865 (100.0%) | 2054 (100.0%) | ＜0.001 | 4196 (100.0%) | 1776 (100.0%) |  |
| No | | 4343 (89.3%) | 1666 (81.1%) |  | 3925 (93.5%) | 1641 (92.4%) |  |
| Yes | | 522 (10.7%) | 388 (18.9%) |  | 271 (6.5%) | 135 (7.6%) | 0.122 |
| Cerebrovascular Disease | | 4865 (100.0%) | 2054 (100.0%) | ＜0.001 | 4196 (100.0%) | 1776 (100.0%) |  |
| No | | 4443 (91.3%) | 1736 (84.5%) |  | 3869 (92.2%) | 1521 (85.6%) |  |
| Yes | | 422 (8.7%) | 318 (15.5%) |  | 327 (7.8%) | 255 (14.4%) | <0.001 |
| Diabetes without Complications | | 4865 (100.0%) | 2054 (100.0%) | 0.002 | 4196 (100.0%) | 1776 (100.0%) |  |
| No | | 3320 (68.2%) | 1324 (64.5%) |  | 2583 (61.6%) | 982 (55.3%) |  |
| Yes | | 1545 (31.8%) | 730 (35.5%) |  | 1613 (38.4%) | 794 (44.7%) | <0.001 |
| Diabetes with Complications | | 4865 (100.0%) | 2054 (100.0%) | ＜0.001 | 4196 (100.0%) | 1776 (100.0%) |  |
| No | | 4269 (87.7%) | 1597 (77.8%) |  | 4196 (100.0%) | 1776 (100.0%) |  |
| Yes | | 596 (12.3%) | 457 (22.2%) |  | 0 (0.0%) | 0 (0.0%) | 1.000 |
| Renal Disease | | 4865 (100.0%) | 2054 (100.0%) | ＜0.001 | 4196 (100.0%) | 1776 (100.0%) |  |
| No | | 4183 (86.0%) | 1412 (68.7%) |  | 3566 (85.0%) | 1276 (71.8%) |  |
| Yes | | 682 (14.0%) | 642 (31.3%) |  | 630 (15.0%) | 500 (28.2%) | <0.001 |
| COPD | | 4865 (100.0%) | 2054 (100.0%) | ＜0.001 | 4196 (100.0%) | 1776 (100.0%) |  |
| No | | 4067 (83.6%) | 1561 (76.0%) |  | 3885 (92.6%) | 1521 (85.6%) |  |
| Yes | | 798 (16.4%) | 493 (24.0%) |  | 311 (7.4%) | 255 (14.4%) | <0.001 |
| Atrial Fibrillation | | 4865 (100.0%) | 2054 (100.0%) | ＜0.001 | 4196 (100.0%) | 1776 (100.0%) |  |
| No | | 3083 (63.4%) | 950 (46.3%) |  | 3871 (92.3%) | 1419 (79.9%) |  |
| Yes | | 1782 (36.6%) | 1104 (53.7%) |  | 325 (7.7%) | 357 (20.1%) | <0.001 |
| Hypertension | | 4865 (100.0%) | 2054 (100.0%) | 0.002 | 4196 (100.0%) | 1776 (100.0%) |  |
| No | | 840 (17.3%) | 291 (14.2%) |  | 1357 (32.3%) | 536 (30.2%) |  |
| Yes | | 4025 (82.7%) | 1763 (85.8%) |  | 2839 (67.7%) | 1240 (69.8%) | 0.108 |
| Chronic Kidney Disease | | 4865 (100.0%) | 2054 (100.0%) | ＜0.001 | 4196 (100.0%) | 1776 (100.0%) |  |
| No | | 4191 (86.1%) | 1414 (68.8%) |  | 3948 (94.1%) | 1626 (91.6%) |  |
| Yes | | 674 (13.9%) | 640 (31.2%) |  | 248 (5.9%) | 150 (8.4%) | <0.001 |
| AKI Stage | | 4862 (99.9%) | 2054 (100.0%) | ＜0.001 | 4196 (100.0%) | 1776 (100.0%) |  |
| 0 | | 4788 (98.4%) | 1974 (96.1%) |  | 3768 (89.8%) | 1361 (76.6%) |  |
| 1 | | 55 (1.1%) | 63 (3.1%) |  | 250 (6.0%) | 210 (11.8%) |  |
| 2 | | 11 (0.2%) | 11 (0.5%) |  | 81 (1.9%) | 98 (5.5%) |  |
| 3 | | 8 (0.2%) | 6 (0.3%) |  | 97 (2.3%) | 107 (6.0%) |  |
| Vasoactive Drug Use | | 4865 (100.0%) | 2054 (100.0%) | ＜0.001 | 4196 (100.0%) | 1776 (100.0%) |  |
| No | | 4071 (83.7%) | 1240 (60.4%) |  | 2464 (58.7%) | 890 (50.1%) |  |
| Yes | | 794 (16.3%) | 814 (39.6%) |  | 1732 (41.3%) | 886 (49.9%) | <0.001 |
| Hemodialysis | | 4865 (100.0%) | 2054 (100.0%) | ＜0.001 | 4196 (100.0%) | 1776 (100.0%) |  |
| No | | 4861 (99.9%) | 2034 (99.0%) |  | 4149 (98.9%) | 1677 (94.4%) |  |
| Yes | | 4 (0.1%) | 20 (1.0%) |  | 47 (1.1%) | 99 (5.6%) | <0.001 |

*Note: In Table S1, the baseline data are presented for the 55 features retained after excluding 9 features with a high missing rate (≥30%). The excluded features and their missing rates are as follows: Albumin (91.9%), Troponin (93.1%), NT-proBNP (99.6%), ALT (86.6%), AST (86.6%), ALP (87.1%), Total Bilirubin (86.5%), LDH (91.2%), and Blood Product Input (76.2%). Abbreviations: AKI, Acute Kidney Injury; COPD, Chronic Obstructive Pulmonary Disease; CCI, Charlson Comorbidity Index; SOFA, Sequential Organ Failure Assessment; SAPS-II, Simplified Acute Physiology Score II; OASIS, Oxford Acute Severity of Illness Score; GCS, Glasgow Coma Scale.*

Table S2. Details of MICE Imputation for Continuous Features.

| **Feature** | **Missing Rate (%)** | **Original Mean** | **Imputed Mean** | **Mean Bias (%)** | **Original Std Dev** | **Imputed Std Dev** | **Std Dev Bias (%)** | **Original Median** | **Imputed Median** | **Median Bias (%)** | **Original Skewness** | **Imputed Skewness** | **KS Statistic** | **KS Test P-Value** | **Distribution Consistency** |
| --- | --- | --- | --- | --- | --- | --- | --- | --- | --- | --- | --- | --- | --- | --- | --- |
| height | 7.72 | 171.813 | 171.756 | 0.03 | 9.59 | 9.537 | 0.55 | 173 | 173 | 0 | -0.377 | -0.352 | 0.0082 | 0.9969 | Passed |
| weight | 1.24 | 89.202 | 89.188 | 0.02 | 18.718 | 18.614 | 0.55 | 87.3 | 87.35 | 0.06 | 0.725 | 0.73 | 0.0035 | 1 | Passed |
| bmi | 7.76 | 30.202 | 30.172 | 0.1 | 5.689 | 5.566 | 2.16 | 29.364 | 29.385 | 0.07 | 1.041 | 1.065 | 0.0195 | 0.3312 | Passed |
| creatinine | 0.14 | 1.09 | 1.09 | 0.03 | 0.972 | 0.971 | 0.07 | 0.9 | 0.9 | 0 | 6.345 | 6.349 | 0.0031 | 1 | Passed |
| bun | 0.14 | 18.779 | 18.775 | 0.02 | 10.642 | 10.635 | 0.07 | 16 | 16 | 0 | 3.252 | 3.255 | 0.0013 | 1 | Passed |
| sodium | 0.31 | 138.347 | 138.347 | 0 | 3.031 | 3.027 | 0.14 | 138 | 138 | 0 | -0.432 | -0.432 | 0.0011 | 1 | Passed |
| potassium | 0.66 | 4.359 | 4.358 | 0.01 | 0.536 | 0.534 | 0.3 | 4.3 | 4.3 | 0 | 0.742 | 0.745 | 0.0021 | 1 | Passed |
| glucose | 2.37 | 6.914 | 6.911 | 0.04 | 2.06 | 2.036 | 1.13 | 6.605 | 6.605 | 0 | 3.458 | 3.498 | 0.0082 | 0.9964 | Passed |
| hemoglobin | 0.14 | 9.694 | 9.696 | 0.02 | 1.861 | 1.862 | 0.02 | 9.6 | 9.6 | 0 | 0.441 | 0.439 | 0.0007 | 1 | Passed |
| platelet | 0.14 | 156.997 | 157.048 | 0.03 | 60.073 | 60.062 | 0.02 | 146 | 146 | 0 | 2.01 | 2.007 | 0.0007 | 1 | Passed |
| wbc | 0.12 | 13.021 | 13.021 | 0 | 6.88 | 6.877 | 0.05 | 12.3 | 12.3 | 0 | 11.759 | 11.762 | 0.0041 | 1 | Passed |
| inr | 0.78 | 1.444 | 1.444 | 0 | 0.395 | 0.394 | 0.25 | 1.4 | 1.4 | 0 | 13.962 | 13.959 | 0.0013 | 1 | Passed |
| ptt | 0.74 | 35.121 | 35.149 | 0.08 | 17.944 | 17.906 | 0.21 | 30.4 | 30.5 | 0.33 | 4.403 | 4.398 | 0.0047 | 1 | Passed |
| lactate | 7.37 | 2.199 | 2.175 | 1.07 | 1.06 | 1.029 | 2.92 | 2 | 2 | 0 | 2.299 | 2.386 | 0.0209 | 0.2546 | Passed |
| ph_24h | 5.16 | 7.393 | 7.393 | 0 | 0.063 | 0.062 | 2.37 | 7.4 | 7.395 | 0.07 | -0.462 | -0.464 | 0.0169 | 0.5055 | Passed |
| fluid_input | 12.08 | 842.861 | 845.721 | 0.34 | 1339.781 | 1312.573 | 2.03 | 265.644 | 302.194 | 13.76 | 2.314 | 2.33 | 0.0456 | 0.0002 | Not passed |
| total_urine_output | 0.93 | 1883.772 | 1876.646 | 0.38 | 907.949 | 909.263 | 0.14 | 1751 | 1747 | 0.23 | 1.154 | 1.143 | 0.005 | 1 | Passed |
| heart_rate | 0.1 | 80.236 | 80.235 | 0 | 10.88 | 10.875 | 0.05 | 80 | 80 | 0 | 0.556 | 0.557 | 0.0005 | 1 | Passed |
| sbp | 0.12 | 113.436 | 113.44 | 0 | 17.447 | 17.439 | 0.05 | 112 | 112 | 0 | 0.457 | 0.457 | 0.0005 | 1 | Passed |
| dbp | 0.12 | 59.245 | 59.245 | 0 | 11.653 | 11.646 | 0.06 | 58 | 58 | 0 | 0.837 | 0.837 | 0.0008 | 1 | Passed |
| mbp | 0.12 | 77.47 | 77.473 | 0 | 13.868 | 13.862 | 0.04 | 76 | 76 | 0 | 2.01 | 2.01 | 0.0006 | 1 | Passed |
| resp_rate | 0.1 | 15.768 | 15.769 | 0.01 | 3.994 | 3.993 | 0.05 | 16 | 16 | 0 | 2.185 | 2.185 | 0.0005 | 1 | Passed |
| temperature | 10.72 | 36.319 | 36.31 | 0.03 | 0.616 | 0.587 | 4.61 | 36.44 | 36.4 | 0.11 | -0.921 | -0.911 | 0.0404 | 0.0011 | Not passed |
| spo2 | 0.12 | 98.894 | 98.893 | 0 | 2.565 | 2.565 | 0.03 | 100 | 100 | 0 | -5.448 | -5.446 | 0.0008 | 1 | Passed |
| invasive_ventilation_hours | 22.07 | 17.269 | 16.588 | 3.94 | 8.518 | 7.893 | 7.33 | 24 | 17.958 | 25.17 | -0.74 | -0.612 | 0.124 | 0 | Not passed |
| bun_creatinine_ratio | 0.14 | 18.89 | 18.89 | 0 | 6.07 | 6.065 | 0.07 | 18.333 | 18.333 | 0 | 1.23 | 1.231 | 0.0034 | 1 | Passed |

Figure S1. Distribution Comparison Plots for MICE Imputation of Continuous Features.

Table S3. Details of Categorical Feature Imputation.

| Feature | Missing Rate (%) | Original Mode | Imputed Mode | Number of Categories | Total Variation Distance (TVD) | Chi-square Statistic | Chi-square P-Value | Distribution Consistency |
| --- | --- | --- | --- | --- | --- | --- | --- | --- |
| insurance | 0.83 | 1 | 1 | 4 | 0.0021 | 0.054 | 0.9967 | Passed |
| gcs_score | 0.31 | 15 | 15 | 13 | 0.0012 | 0.0139 | 1 | Passed |
| aki_stage | 0.06 | 0 | 0 | 4 | 0 | 0 | 1 | Passed |

Figure S2. Comparison Plots for Categorical Feature Imputation.

Figure S3. Coefficient Path Plot for the Elastic Net Regression.

Figure S4. Feature Selection State Evolution and Importance Convergence Plot for the Boruta Algorithm.

Figure S5. Flowchart of the Dual Feature Selection Strategy (Elastic Net and Boruta) Integrated via Borda Count.

Table S4. Detailed Results of Paired t-Test Comparisons Among the Nine Models (uncorrected p-values, with α = 0.05). Multiplicity-corrected results — including Bonferroni-adjusted p-values and Benjamini–Hochberg FDR-adjusted q-values — are reported in Table S9

| Modle1 | Modle1 CV AUC | Modle2 | Modle2 CV AUC | P-Value |
| --- | --- | --- | --- | --- |
| LR | 0.7617 | RF | 0.7567 | 0.1741 |
| LR | 0.7617 | SVM | 0.7604 | 0.4961 |
| LR | 0.7617 | KNN | 0.7242 | 0.0002 |
| LR | 0.7617 | Decision Tree | 0.6639 | 0.001 |
| LR | 0.7617 | XGBoost | 0.7562 | 0.6429 |
| LR | 0.7617 | LightGBM | 0.7582 | 0.2821 |
| LR | 0.7617 | CatBoost | 0.7648 | 0.1295 |
| LR | 0.7617 | DNN | 0.7598 | 0.346 |
| RF | 0.7567 | SVM | 0.7604 | 0.3369 |
| RF | 0.7567 | KNN | 0.7242 | 0.001 |
| RF | 0.7567 | Decision Tree | 0.6639 | 0.001 |
| RF | 0.7567 | XGBoost | 0.7562 | 0.9653 |
| RF | 0.7567 | LightGBM | 0.7582 | 0.4509 |
| RF | 0.7567 | CatBoost | 0.7648 | 0.0083 |
| RF | 0.7567 | DNN | 0.7598 | 0.4055 |
| SVM | 0.7604 | KNN | 0.7242 | 0.001 |
| SVM | 0.7604 | Decision Tree | 0.6639 | 0.001 |
| SVM | 0.7604 | XGBoost | 0.7562 | 0.7113 |
| SVM | 0.7604 | LightGBM | 0.7582 | 0.4793 |
| SVM | 0.7604 | CatBoost | 0.7648 | 0.0916 |
| SVM | 0.7604 | DNN | 0.7598 | 0.7652 |
| KNN | 0.7242 | Decision Tree | 0.6639 | 0.001 |
| KNN | 0.7242 | XGBoost | 0.7562 | 0.0201 |
| KNN | 0.7242 | LightGBM | 0.7582 | 0.001 |
| KNN | 0.7242 | CatBoost | 0.7648 | 0.001 |
| KNN | 0.7242 | DNN | 0.7598 | 0.001 |
| Decision Tree | 0.6639 | XGBoost | 0.7562 | 0.0007 |
| Decision Tree | 0.6639 | LightGBM | 0.7582 | 0.001 |
| Decision Tree | 0.6639 | CatBoost | 0.7648 | 0.001 |
| Decision Tree | 0.6639 | DNN | 0.7598 | 0.001 |
| XGBoost | 0.7562 | LightGBM | 0.7582 | 0.8697 |
| XGBoost | 0.7562 | CatBoost | 0.7648 | 0.4939 |
| XGBoost | 0.7562 | DNN | 0.7598 | 0.7495 |
| LightGBM | 0.7582 | CatBoost | 0.7648 | 0.0063 |
| LightGBM | 0.7582 | DNN | 0.7598 | 0.5757 |
| CatBoost | 0.7648 | DNN | 0.7598 | 0.0375 |

Figure S6. A Low-Risk Patient with Non-Prolonged ICU Stay (Lowest Predicted Probability: 0.057).

Figure S6 represents a patient in the internal validation dataset who actually did not experience a prolonged ICU stay and had the lowest predicted risk of prolonged stay (probability: 0.057). The features contributing to their "rapid ICU discharge" can be identified in descending order of impact: a CCI of 0 points, a fluid input of 181 ml within 24 hours, normal SAPS, SOFA, and GCS scores, no history of atrial fibrillation or heart failure, and no use of vasoactive drugs. In the SHAP analysis, blue features indicate a reduced risk of postoperative prolonged ICU stay, while red features indicate an increased risk.

Figure S7. A High-Risk Patient with Prolonged ICU Stay (Highest Predicted Probability: 0.945).

Figure S7 represents a patient in the internal validation dataset who actually experienced a prolonged ICU stay and had the highest predicted risk for it (probability: 0.945). It can be observed that all factors for this patient are unfavorable. Particularly significant contributors are the poor SOFA score, high fluid input (2306 ml), a very poor CCI, and the use of vasoactive drugs. In the SHAP analysis, blue features indicate a reduced risk of postoperative prolonged ICU stay, while red features indicate an increased risk.

Figure S8. SHAP Dependence Plot - Fluid Input within First 24 Hours of ICU Admission

Figure S9. SHAP Dependence Plot - Charlson Comorbidity Index (CCI)

Figure S10. SHAP Dependence Plot - History of Atrial Fibrillation

Figure S11. SHAP Dependence Plot - Simplified Acute Physiology Score II (SAPS-II)

Figure S12. SHAP Dependence Plot - SOFA Score

Figure S13. SHAP Dependence Plot - Vasopressor Use

Figure S14. SHAP Dependence Plot - History of Congestive Heart Failure

Figure S15. SHAP Dependence Plot - Glasgow Coma Scale (GCS) Score

Figure S16. Screenshot of the Online Risk Prediction Calculator.

**Supplementary Material 7.** Additional Sensitivity and Methodological Tables Supporting the Revision

The following Tables (S5–S14) and Figures (S17, S18) provide the full numerical support for the methodological revisions described in the Response to Reviewers. Each table is identified by the corresponding reviewer comment and the underlying analysis script archived at Zenodo with permanent DOI 10.5281/zenodo.20135180 (https://doi.org/10.5281/zenodo.20135180).

Table S5. Three-strategy imputation sensitivity analysis (internal MIMIC-IV test set + external eICU-CRD 2.0 cohort). Sample sizes differ across strategies by design: the complete-case strategy excludes any patient with a missing value in the eight modelling features (n_train = 4,279; n_test = 1,796; n_external = 5,305 after dropping cases with missing SAPS-II), whereas the PMM-style and missForest strategies retain all eligible patients (n_train = 4,843; n_test = 2,076; n_external = 5,972) because missing values are filled by imputation. This is the methodologically expected behaviour of complete-case analysis.

| Imputation strategy | MIMIC train n | MIMIC test n | Internal AUC (95% CI) | External n | External AUC (95% CI) | Δ external vs primary 0.6452 |
| --- | --- | --- | --- | --- | --- | --- |
| Complete-case | 4279 | 1796 | 0.7359 (0.7094–0.7604) | 5305 | 0.5927 (0.5759–0.6099) | -0.0525 |
| PMM (m=5, pooled) | 4843 | 2076 | 0.7421 (0.7193–0.7659) | 5972 | 0.6097 (0.5960–0.6240) | -0.0355 |
| missForest (single) | 4843 | 2076 | 0.7406 (0.7200–0.7644) | 5972 | 0.6058 (0.5919–0.6197) | -0.0394 |

Table S6. SHAP top-3 features for the primary deployed CatBoost model and the three alternative imputation strategies. Top-1 (fluid intake) and top-2 (Charlson Comorbidity Index) ranks are preserved across all four model variants. The third-ranked feature is atrial fibrillation in the primary deployed model (mean |SHAP| = 0.2046) and SAPS-II in each of the three alternative imputation strategies (mean |SHAP| = 0.2936–0.3057), consistent with their near-tied SHAP importance in the primary analysis (|SHAP| 0.205 vs 0.202; difference < 1.5 % of the top-1 magnitude).

| Strategy | Rank | Feature | Mean \|SHAP\| |
| --- | --- | --- | --- |
| Primary (deployed CatBoost) | 1 | fluid_input | 0.4458 |
| Primary (deployed CatBoost) | 2 | charlson_comorbidity_index | 0.3268 |
| Primary (deployed CatBoost) | 3 | atrial_fibrillation | 0.2046 |
| Complete-case | 1 | fluid_input | 0.4904 |
| Complete-case | 2 | charlson_comorbidity_index | 0.3434 |
| Complete-case | 3 | sapsii_score | 0.2954 |
| PMM (m=5, pooled) | 1 | fluid_input | 0.4732 |
| PMM (m=5, pooled) | 2 | charlson_comorbidity_index | 0.3378 |
| PMM (m=5, pooled) | 3 | sapsii_score | 0.2936 |
| missForest (single) | 1 | fluid_input | 0.4794 |
| missForest (single) | 2 | charlson_comorbidity_index | 0.3336 |
| missForest (single) | 3 | sapsii_score | 0.3057 |

Table S7. Three-window CatBoost performance at 6-h, 12-h, and 24-h feature extraction windows (internal MIMIC-IV test set + external eICU-CRD 2.0 cohort). Provides exploratory full-pipeline confirmation of the per-patient hourly trajectory analysis (Figure S17). Specification described in main-text Methods Section 1.2.

| Window | MIMIC train n | CV-10 AUC (mean ± SD) | Internal test AUC | External eICU n | External AUC (uncalibrated, 95% CI) | fluid_input median (MIMIC / eICU) |
| --- | --- | --- | --- | --- | --- | --- |
| 6h | 4843 | 0.7357 ± 0.0173 | 0.7357 | 5972 | 0.5924 (0.5778–0.6075) | 25.2 mL / 0.0 mL |
| 12h | 4843 | 0.7423 ± 0.0215 | 0.7477 | 5972 | 0.6111 (0.5955–0.6267) | 100.3 mL / 305.0 mL |
| 24h | 4843 | 0.7527 ± 0.0268 | 0.7581 | 5972 | 0.6139 (0.5993–0.6290) | 199.7 mL / 862.5 mL |

Table S8. Per-hour cumulative fluid_input median by outcome and Mann–Whitney one-sided test (n = 6,919 MIMIC-IV training cohort). At ICU hours 0–6 the two outcome groups are clinically indistinguishable (median difference 2.2 mL at 6 h); the groups separate sharply between hours 12 and 24. Provides direct empirical evidence on the reverse-causation question. Extraction specification described in main-text Methods Section 1.2.

| Hour from ICU intime | Prolonged median (mL) | Non-prolonged median (mL) | Median difference (mL) | Mann–Whitney U | p (one-sided) |
| --- | --- | --- | --- | --- | --- |
| 1 | 0.0 | 0.0 | +0.0 | 5157220 | 5.60e-09 |
| 2 | 0.0 | 0.0 | +0.0 | 5235120 | 1.31e-08 |
| 3 | 0.0 | 0.0 | +0.0 | 5138030 | 0.0033 |
| 4 | 0.0 | 0.0 | +0.0 | 5055500 | 0.1186 |
| 5 | 10.3 | 15.1 | -4.8 | 5101010 | 0.0407 |
| 6 | 26.8 | 24.6 | +2.2 | 5228500 | 3.17e-04 |
| 7 | 46.2 | 35.1 | +11.1 | 5344780 | 3.90e-07 |
| 8 | 66.0 | 46.2 | +19.8 | 5483760 | 6.45e-12 |
| 9 | 87.9 | 57.6 | +30.3 | 5578560 | 5.29e-16 |
| 10 | 114.3 | 67.9 | +46.4 | 5636250 | 8.13e-19 |
| 11 | 137.2 | 77.9 | +59.2 | 5732180 | 4.66e-24 |
| 12 | 158.7 | 88.8 | +69.8 | 5781130 | 5.34e-27 |
| 13 | 181.1 | 97.7 | +83.5 | 5825140 | 8.47e-30 |
| 14 | 212.4 | 105.0 | +107.4 | 5883110 | 1.04e-33 |
| 15 | 245.4 | 112.5 | +133.0 | 5935610 | 1.80e-37 |
| 16 | 277.4 | 119.9 | +157.5 | 5972440 | 3.10e-40 |
| 17 | 307.3 | 126.4 | +180.9 | 6002690 | 1.40e-42 |
| 18 | 331.7 | 133.7 | +198.0 | 6007100 | 6.27e-43 |
| 19 | 355.6 | 140.6 | +214.9 | 6035800 | 3.15e-45 |
| 20 | 378.7 | 146.8 | +231.9 | 6065960 | 1.03e-47 |
| 21 | 394.7 | 152.0 | +242.6 | 6086040 | 2.11e-49 |
| 22 | 414.7 | 155.3 | +259.5 | 6114000 | 8.30e-52 |
| 23 | 443.0 | 157.7 | +285.3 | 6145610 | 1.34e-54 |
| 24 | 452.1 | 160.9 | +291.2 | 6169460 | 9.36e-57 |

Table S9. Multiple-testing correction for the 36 pairwise model comparisons. Reports the t-statistic, the originally rounded p-value (manuscript Table 1), the exact p-value recomputed from the t-statistic against 10-fold cross-validation degrees of freedom (df = 9), the Bonferroni-adjusted p-value (per-test threshold ≈ 0.0014), and the Benjamini–Hochberg adjusted q-value.

| Model A | Model B | AUC A | AUC B | t | p (rounded) | p (exact) | Bonferroni p | BH-FDR q |
| --- | --- | --- | --- | --- | --- | --- | --- | --- |
| Logistic Regression | Random Forest | 0.7617 | 0.7567 | 1.476 | 0.1741 | 0.1741 | 1 | 0.2984 |
| Logistic Regression | SVM | 0.7617 | 0.7604 | 0.709 | 0.4961 | 0.4961 | 1 | 0.6159 |
| Logistic Regression | KNN | 0.7617 | 0.7242 | 6.009 | 0.0002 | 0.0002003 | 0.007211 | 0.0005547 |
| Logistic Regression | Decision Tree | 0.7617 | 0.6639 | 9.496 | 0.0000 | 5.496e-06 | 0.0001978 | 3.297e-05 |
| Logistic Regression | XGBoost | 0.7617 | 0.7562 | 0.480 | 0.6429 | 0.6429 | 1 | 0.7466 |
| Logistic Regression | LightGBM | 0.7617 | 0.7582 | 1.144 | 0.2821 | 0.2821 | 1 | 0.4616 |
| Logistic Regression | CatBoost | 0.7617 | 0.7648 | -1.669 | 0.1295 | 0.1295 | 1 | 0.2331 |
| Logistic Regression | Deep Neural Network | 0.7617 | 0.7598 | 0.994 | 0.3460 | 0.3461 | 1 | 0.5191 |
| Random Forest | SVM | 0.7567 | 0.7604 | -1.014 | 0.3369 | 0.3368 | 1 | 0.5191 |
| Random Forest | KNN | 0.7567 | 0.7242 | 7.454 | 0.0000 | 3.876e-05 | 0.001395 | 0.000155 |
| Random Forest | Decision Tree | 0.7567 | 0.6639 | 10.902 | 0.0000 | 1.736e-06 | 6.249e-05 | 2.083e-05 |
| Random Forest | XGBoost | 0.7567 | 0.7562 | 0.045 | 0.9653 | 0.9653 | 1 | 0.9653 |
| Random Forest | LightGBM | 0.7567 | 0.7582 | -0.788 | 0.4509 | 0.451 | 1 | 0.6159 |
| Random Forest | CatBoost | 0.7567 | 0.7648 | -3.364 | 0.0083 | 0.008339 | 0.3002 | 0.01876 |
| Random Forest | Deep Neural Network | 0.7567 | 0.7598 | -0.873 | 0.4055 | 0.4056 | 1 | 0.584 |
| SVM | KNN | 0.7604 | 0.7242 | 6.460 | 0.0001 | 0.0001167 | 0.004201 | 0.0003951 |
| SVM | Decision Tree | 0.7604 | 0.6639 | 9.801 | 0.0000 | 4.228e-06 | 0.0001522 | 3.044e-05 |
| SVM | XGBoost | 0.7604 | 0.7562 | 0.382 | 0.7113 | 0.7113 | 1 | 0.8002 |
| SVM | LightGBM | 0.7604 | 0.7582 | 0.738 | 0.4793 | 0.4794 | 1 | 0.6159 |
| SVM | CatBoost | 0.7604 | 0.7648 | -1.889 | 0.0916 | 0.09156 | 1 | 0.1735 |
| SVM | Deep Neural Network | 0.7604 | 0.7598 | 0.308 | 0.7652 | 0.7652 | 1 | 0.8103 |
| KNN | Decision Tree | 0.7242 | 0.6639 | 6.302 | 0.0001 | 0.0001407 | 0.005066 | 0.0004221 |
| KNN | XGBoost | 0.7242 | 0.7562 | -2.818 | 0.0201 | 0.02012 | 0.7242 | 0.0426 |
| KNN | LightGBM | 0.7242 | 0.7582 | -8.392 | 0.0000 | 1.507e-05 | 0.0005426 | 7.752e-05 |
| KNN | CatBoost | 0.7242 | 0.7648 | -7.832 | 0.0000 | 2.621e-05 | 0.0009436 | 0.000118 |
| KNN | Deep Neural Network | 0.7242 | 0.7598 | -6.431 | 0.0001 | 0.0001207 | 0.004346 | 0.0003951 |
| Decision Tree | XGBoost | 0.6639 | 0.7562 | -5.085 | 0.0007 | 0.0006587 | 0.02371 | 0.001694 |
| Decision Tree | LightGBM | 0.6639 | 0.7582 | -11.248 | 0.0000 | 1.334e-06 | 4.802e-05 | 2.083e-05 |
| Decision Tree | CatBoost | 0.6639 | 0.7648 | -11.140 | 0.0000 | 1.447e-06 | 5.208e-05 | 2.083e-05 |
| Decision Tree | Deep Neural Network | 0.6639 | 0.7598 | -9.890 | 0.0000 | 3.922e-06 | 0.0001412 | 3.044e-05 |
| XGBoost | LightGBM | 0.7562 | 0.7582 | -0.169 | 0.8697 | 0.8697 | 1 | 0.8945 |
| XGBoost | CatBoost | 0.7562 | 0.7648 | -0.713 | 0.4939 | 0.4939 | 1 | 0.6159 |
| XGBoost | Deep Neural Network | 0.7562 | 0.7598 | -0.329 | 0.7495 | 0.7495 | 1 | 0.8103 |
| LightGBM | CatBoost | 0.7582 | 0.7648 | -3.543 | 0.0063 | 0.006284 | 0.2262 | 0.01508 |
| LightGBM | Deep Neural Network | 0.7582 | 0.7598 | -0.581 | 0.5757 | 0.5756 | 1 | 0.6908 |
| CatBoost | Deep Neural Network | 0.7648 | 0.7598 | 2.438 | 0.0375 | 0.03752 | 1 | 0.07504 |

Table S10. Coefficients of the parsimony-preferring Logistic Regression secondary calculator. Trained on the same MIMIC-IV 7:3 stratified split as the primary CatBoost model (random_state = 42); GridSearchCV-selected hyper-parameters: penalty = L1, C = 1, solver = liblinear. Coefficients are reported on the standardised feature scale; the per-feature scaler mean (μ) and standard deviation (σ) are provided so the linear predictor η = β₀ + Σ βᵢ × (xᵢ − μᵢ) / σᵢ can be reproduced exactly. The predicted probability of prolonged ICU LOS is p = 1 / (1 + exp(−η)). Released alongside the deployed CatBoost calculator for users preferring a directly-inspectable linear model. Performance metrics of the LR sibling (CV-10 AUC 0.7632 ± 0.0155; internal-test AUC 0.7356; external AUC 0.6227) are reported in the main-text Discussion (Selection of the Deployed Model). Patient-level external predictions and a usage README are archived alongside this coefficient table on Zenodo with permanent DOI 10.5281/zenodo.20135180 (https://doi.org/10.5281/zenodo.20135180).

| Feature | Standardised coefficient | Scaler mean (μ) | Scaler SD (σ) |
| --- | --- | --- | --- |
| sofa_score | 0.3243 | 5.128 | 2.726 |
| charlson_comorbidity_index | 0.2824 | 4.579 | 2.264 |
| fluid_input | 0.3857 | 2132.020 | 5982.260 |
| vasopressor_use | 0.2125 | 0.234 | 0.423 |
| sapsii_score | 0.4876 | 38.208 | 11.672 |
| congestive_heart_failure | 0.1652 | 0.258 | 0.437 |
| gcs_score | 0.4523 | 13.358 | 3.625 |
| atrial_fibrillation | 0.2045 | 0.418 | 0.493 |
| (intercept) | -1.0225 |  |  |

Table S11. Decision Curve Analysis net benefit grid at thresholds t = 0.10–0.50 on the external eICU-CRD 2.0 cohort (n = 5,972). Reported under the prevalence-anchored threshold framing.

| Threshold (t) | NB recalibrated | NB uncalibrated | NB treat-all | NB treat-none |
| --- | --- | --- | --- | --- |
| 0.100 | 0.2193 | 0.2193 | 0.2193 | 0.0000 |
| 0.125 | 0.1973 | 0.1975 | 0.1970 | 0.0000 |
| 0.150 | 0.1753 | 0.1731 | 0.1734 | 0.0000 |
| 0.175 | 0.1536 | 0.1512 | 0.1483 | 0.0000 |
| 0.200 | 0.1309 | 0.1305 | 0.1217 | 0.0000 |
| 0.225 | 0.1122 | 0.1137 | 0.0934 | 0.0000 |
| 0.250 | 0.0983 | 0.0953 | 0.0632 | 0.0000 |
| 0.275 | 0.0791 | 0.0796 | 0.0309 | 0.0000 |
| 0.300 | 0.0589 | 0.0611 | 0.0000 | 0.0000 |
| 0.325 | 0.0432 | 0.0453 | 0.0000 | 0.0000 |
| 0.350 | 0.0317 | 0.0325 | 0.0000 | 0.0000 |
| 0.375 | 0.0229 | 0.0199 | 0.0000 | 0.0000 |
| 0.400 | 0.0181 | 0.0122 | 0.0000 | 0.0000 |
| 0.425 | 0.0127 | 0.0032 | 0.0000 | 0.0000 |
| 0.450 | 0.0079 | -0.0006 | 0.0000 | 0.0000 |
| 0.475 | 0.0063 | -0.0042 | 0.0000 | 0.0000 |
| 0.500 | 0.0033 | -0.0084 | 0.0000 | 0.0000 |

Table S12. Operating-point characteristics at the prevalence-anchored thresholds 0.20, 0.25, 0.30, 0.35, and 0.40 on the external eICU-CRD 2.0 cohort. Recalibrated and uncalibrated rows are reported in parallel.

| Model | Threshold | n flagged | Sensitivity | Specificity | PPV | NPV | F1 | Accuracy |
| --- | --- | --- | --- | --- | --- | --- | --- | --- |
| recalibrated | 0.20 | 4879 | 0.901 | 0.219 | 0.328 | 0.840 | 0.481 | 0.422 |
| recalibrated | 0.25 | 3579 | 0.752 | 0.465 | 0.373 | 0.816 | 0.499 | 0.550 |
| recalibrated | 0.30 | 2446 | 0.552 | 0.651 | 0.401 | 0.774 | 0.464 | 0.621 |
| recalibrated | 0.35 | 1666 | 0.398 | 0.771 | 0.424 | 0.751 | 0.410 | 0.660 |
| recalibrated | 0.40 | 1073 | 0.278 | 0.862 | 0.460 | 0.738 | 0.347 | 0.688 |
| uncalibrated | 0.20 | 4153 | 0.819 | 0.357 | 0.350 | 0.823 | 0.490 | 0.494 |
| uncalibrated | 0.25 | 3300 | 0.705 | 0.512 | 0.379 | 0.804 | 0.493 | 0.569 |
| uncalibrated | 0.30 | 2558 | 0.576 | 0.634 | 0.400 | 0.779 | 0.472 | 0.617 |
| uncalibrated | 0.35 | 2037 | 0.472 | 0.715 | 0.412 | 0.762 | 0.440 | 0.642 |
| uncalibrated | 0.40 | 1628 | 0.391 | 0.778 | 0.427 | 0.751 | 0.408 | 0.663 |

Table S13. Cutpoint sensitivity analysis comparing the primary >3-day cutpoint with the >5-day clinical-convention cutpoint. Both columns reflect independently retrained models using a simplified training pipeline (default CatBoost hyperparameters, fixed random seed) for direct cutpoint comparison; deployed-model headline metrics reported in main-text Results Section 2.5 apply only to the primary >3-day analysis with full Optuna Bayesian hyperparameter optimisation, hence the deployed model achieves higher discrimination (internal AUC 0.7739; external AUC 0.6452) than the >3-day column shown here.

| Metric | >3 days | >5 days |
| --- | --- | --- |
| MIMIC train n | 4832 | 4832 |
| MIMIC test n | 2071 | 2071 |
| eICU external n | 5972 | 5972 |
| MIMIC training prevalence | 0.297 | 0.118 |
| eICU external prevalence | 0.297 | 0.127 |
| CV-10 AUC mean | 0.7572 | 0.8273 |
| CV-10 AUC SD | 0.0175 | 0.0333 |
| Internal test AUC | 0.7522 | 0.7895 |
| External AUC (uncalibrated) | 0.5909 | 0.6066 |
| External AUC (recalibrated) | 0.5907 | 0.6060 |

Table S14a. Variance Inflation Factors (VIF) for the eight deployed features (after MICE imputation). All VIF values are below the conventional cutoff of 5 for problematic multicollinearity.

| Feature | VIF |
| --- | --- |
| sofa_score_24h | 1.779 |
| charlson_comorbidity_index | 1.643 |
| fluid_input_24h | 1.083 |
| vasopressor_use | 1.136 |
| sapsii_score | 3.172 |
| congestive_heart_failure | 1.357 |
| gcs_score_24h | 2.242 |
| atrial_fibrillation | 1.065 |

Table S14b. Reduced-model comparison: scores-only (SOFA / CCI / SAPS-II / GCS) vs raw-only (fluid_input / vasopressor / CHF / AF) vs full eight-feature CatBoost. Both reduced models perform substantially worse than the full specification, indicating empirical complementarity rather than redundancy. Note: absolute AUCs in this table are computed under the configuration applied uniformly across the cohort/window/imputation sensitivity analyses reported elsewhere in this Supplementary; they are internally comparable across the three model variants but are not directly comparable in absolute magnitude to the principal external AUC of 0.6452 reported in Section 2.5.

| Model | n features | CV-10 AUC ± SD (training) | Internal AUC (95% CI) | External AUC (95% CI) |
| --- | --- | --- | --- | --- |
| full_8 | 8 | 0.7527 ± 0.0268 | 0.7581 (0.7357–0.7809) | 0.6167 (0.6020–0.6315) |
| scores_only_4 | 4 | 0.7189 ± 0.0260 | 0.7222 (0.6971–0.7442) | 0.6007 (0.5845–0.6162) |
| raw_only_4 | 4 | 0.7041 ± 0.0185 | 0.7161 (0.6923–0.7416) | 0.5941 (0.5790–0.6085) |

Figure S17. Per-patient hourly cumulative fluid_input by ICU LOS outcome (MIMIC-IV training cohort, n = 6,919). Median trajectories with interquartile band, stratified by the prolonged-stay outcome (>3 days). Vertical dotted lines mark hours 6, 12, and 24. The two groups are clinically indistinguishable at h ≤ 6 (median difference 2.2 mL at 6 hours) and separate sharply between 12 and 24 hours (median difference 291.2 mL at 24 hours). Extraction specification described in main-text Methods Section 1.2.

Figure S18. Bonferroni-corrected heatmap of paired t-test comparisons among the nine prediction models (training cohort, 10-fold cross-validation). Per-test threshold = 0.05 / 36 ≈ 0.0014. The Bonferroni-corrected panel is shown alongside the uncorrected heatmap in main-text Figure 3 and is reproduced here so that the uncorrected Table S4, multiplicity-corrected Table S9, and the corrected heatmap form a self-contained block on multiple-testing control. Asterisks (*) indicate comparisons with Bonferroni-adjusted p < 0.05.
